# Evaluation of quantum dot conjugated antibodies for immunofluorescent labelling of cellular targets

**DOI:** 10.3762/bjnano.8.125

**Published:** 2017-06-09

**Authors:** Jennifer E Francis, David Mason, Raphaël Lévy

**Affiliations:** 1 Department of Biochemistry, Institute of Integrative Biology, Biosciences Building, Crown Street, Liverpool, L69 7ZB, United Kingdom; 2 Centre for Cell Imaging, Institute of Integrative Biology, Biosciences Building, Crown Street, Liverpool, L69 7ZB, United Kingdom

**Keywords:** imaging, immunofluorescence, microscopy, nanoparticles, quantum dots

## Abstract

Semiconductor quantum dots (Qdots) have been utilised as probes in fluorescence microscopy and provide an alternative to fluorescent dyes and fluorescent proteins due to their brightness, photostability, and the possibility to excite different Qdots with a single wavelength. In spite of these attractive properties, their implemenation by biologists has been somewhat limited and only a few Qdot conjugates are commercially available for the labelling of cellular targets. Although many protocols have been reported for the specific labelling of proteins with Qdots, the majority of these relied on Qdot-conjugated antibodies synthesised specifically by the authors (and therefore not widely available), which limits the scope of applications and complicates replication. Here, the specificity of a commercially available, Qdot-conjugated secondary antibody (Qdot-Ab) was tested against several primary IgG antibodies. The antigens were labelled simultaneously with a fluorescent dye coupled to a secondary antibody (Dye-Ab) and the Qdot-Ab. Although, the Dye-Ab labelled all of the intended target proteins, the Qdot-Ab was found bound to only some of the protein targets in the cytosol and could not reach the nucleus, even after extensive cell permeabilisation.

## Introduction

Quantum dots (Qdots) are nanometre-sized semiconductor nanocrystals that typically consist of a metallic core of cadmium selenium (CdSe) and an inorganic zinc sulfide (ZnS) shell and have been applied as fluorescent probes for the labelling of biological structures [1–2]. To make Qdots water soluble, and thus suitable for biological applications, their surface is modified either by coating with hydrophilic ligands (such as poly(ethylene glycol) (PEG) [3–4]) or they are encapsulated in amphiphilic polymers [5]. Antibodies that recognise specific biological targets can then be conjugated to these Qdots for use in immunofluorescence. Conventional immunocytochemistry (ICC) protocols involve the chemical fixation of cells, followed by permeabilisation with a detergent. This creates pores in the cell membrane, allowing primary and secondary antibodies to gain access to the protein of interest.

Qdots are an attractive alternative to traditional fluorescent dyes for ICC because they are much brighter and more photostable [6–7]. In contrast to fluorescent dyes, Qdots can be excited with a wide range of wavelengths and have narrow emission spectra, which is advantageous for multiplex imaging [8–9]. The emission maxima of Qdots are dependent on their size; the emission peak for large Qdots is in the red end of the spectra and smaller Qdots in the blue region [1]. Qdots are also an ideal probe choice for super-resolution imaging techniques that require stochastic optical fluctuation, as they exhibit well-characterised blinking between fluorescent and non-fluorescent states [10–11].

Despite these favourable characteristics, the overall hydrodynamic radius of a Qdot (15–20 nm) is much larger than that of a fluorescent dye molecule [12–14]. As a result, one large Qdot may host many antibodies, whereas many fluorescent dye molecules can be coupled to a single antibody [9,15]. Furthermore, the overall size of commercially available Qdots is further enlarged by the addition of protective layers to maintain stability and shelf life [13,16]. Qdot-conjugated antibodies (Qdot-Abs) are therefore unlikely to replace fluorescent dyes in ICC due to their inferior penetration capability [17–18]. Despite the assumption that manufactured Qdots are quality controlled, the considerable batch-to-batch variability means that each time a new lot is purchased, the labelling conditions need to be optimised [19]. The use of commercial Qdot-Abs is also expensive, as they are used in quantities on the order of 20 nM, yet are supplied at a higher price per unit volume compared to fluorescent dyes [16,20–21]. Despite having been around for several decades, Qdots are rarely used in routine ICC [1].

There are some notable examples of Qdot-Abs in the published literature where they have been used to label glycine receptors [22], glial fibrillary acidic proteins (GFAPs) [12], mortalin [23], erythrocytes [24], GRP78 protein [25], caveolin-1 [26], golgi [20], and nuclear HER2 targets [6]. The majority of this labelling, however, was done with in-house synthesised Qdots rather than commercially available Qdots [27]. The use of commercially available Qdots allows for the controlled synthesis and thus the size of the Qdot-Abs can be kept to a minimum. However, it has been noted that specific labelling of nuclear and some cytoplasmic structures with Qdot-Abs is not always reproducible [1,6,13,20,28]. Another concern amongst users of commercial Qdot-Abs is non-specific labelling and the formation of aggregates, which may introduce artifacts and lead to misinterpretation of false positive results [12–13].

**Figure 1:**
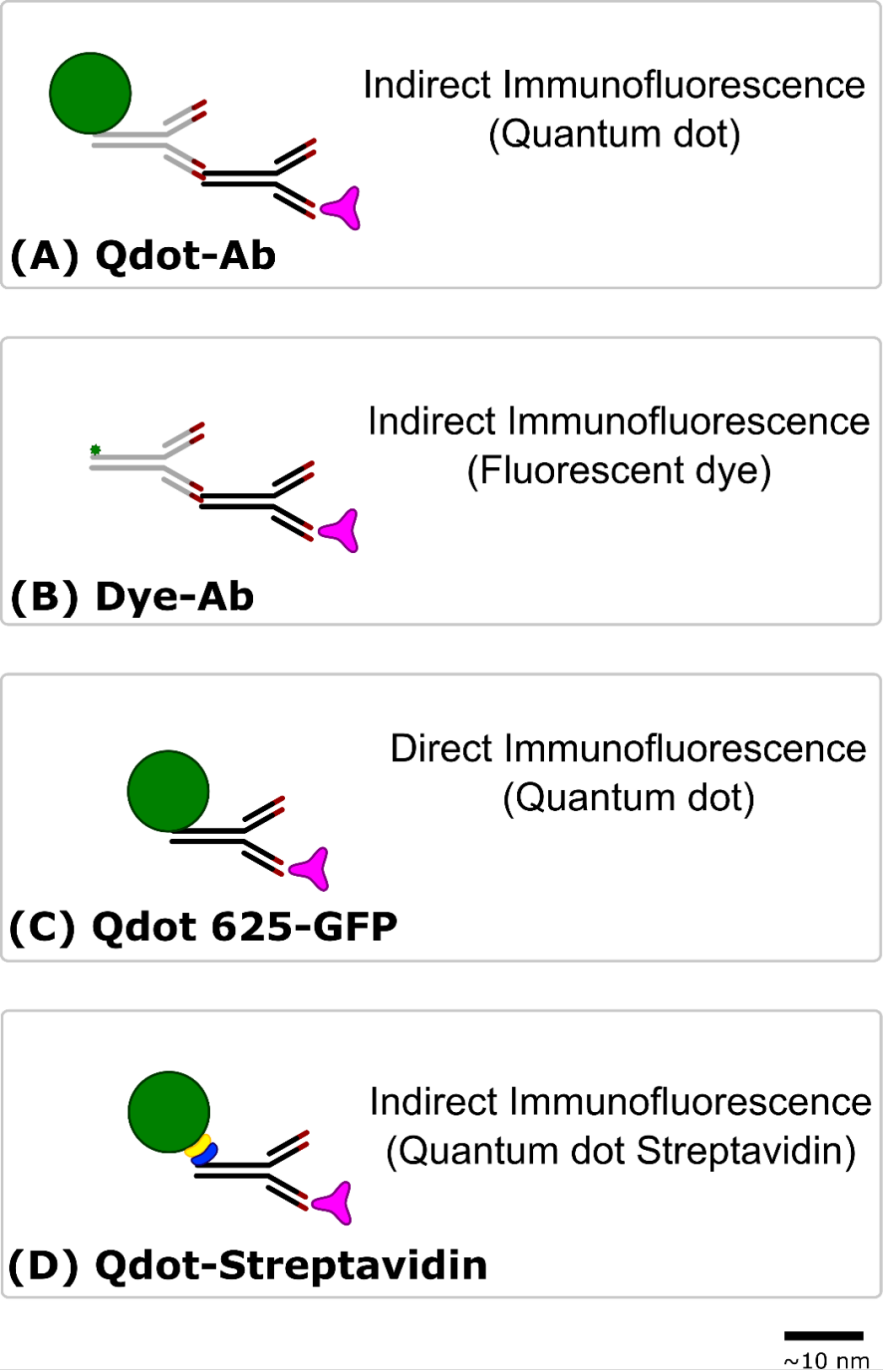
Strategies for immunofluorescence labelling. A fluorescent label (green) is conjugated to a secondary antibody (grey) or directly to a primary antibody (black), containing antigen-binding sites (red), which recognises and binds to a specific antigen (pink). Immunofluorescence labelling was either indirect with a primary antibody and a Qdot 625/Qdot 525 (Qdot-Ab) (A) or fluorescent dye such as Alexa Fluor 488/Cyanine 3 (Dye-Ab) (B) conjugated to a secondary antibody, or direct with an anti-GFP primary antibody conjugated to Qdot 625 (Qdot 625-GFP) (C). An alternative Qdot 625 was tried for indirect immunofluorescence labelling using a Qdot 625 streptavidin (yellow) conjugate (Qdot-Streptavidin) and biotinylated (blue) primary antibody (D). Scale bar is 10 nm.

Here, we focus on the specificity of commercially available Qdot 625 conjugated antibodies (Thermo Fisher Scientific, UK), with an emission maxima of 625 nm (excitation and emission spectra available in Figure S1 in Supporting Information File 1), in fixed cells (Figure 1). Different protein targets were labelled simultaneously with both a secondary antibody conjugated to a fluorescent dye (Dye-Ab) and a Qdot 625 conjugated secondary antibody (Qdot 625-Ab). A Qdot 525 conjugated secondary antibody (Qdot 525-Ab) was also evaluated, with an emission maxima of 525 nm (excitation and emission spectra available in Supporting Information File 1, Figure S2); along with an anti-GFP Qdot 625 conjugate (Qdot 625-GFP), anti-tubulin Qdot 625 conjugate (Qdot 625-Tubulin), and Qdot 625 conjugated to streptavidin (Qdot-Streptavidin). We found that while the prototypical target of Qdot-Abs: tubulin, could be easily labelled, several other protein targets including nuclear proteins and components of large cytosolic protein complexes could not be labelled with Qdot-Abs. We posit that this may be due to steric hindrance associated with the size of the Qdot-Abs.

## Results and Discussion

**Figure 2:**
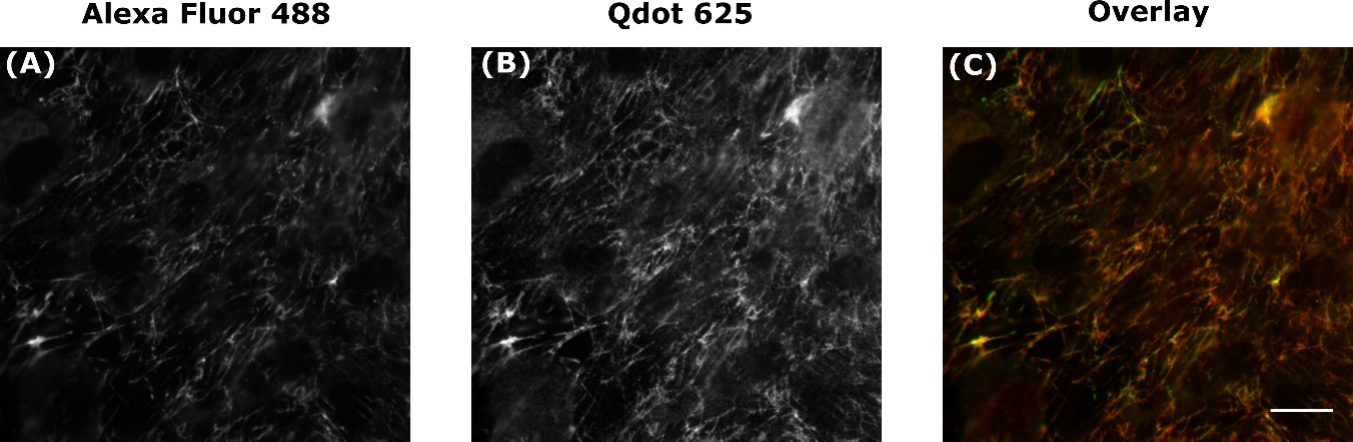
Specific labelling of fibronectin with Qdots. Fixed rat mammary (Rama) 27 fibroblasts were dual labelled with green Alexa Fluor 488 (A) and a red Qdot 625 (B) to produce an overlaid wide-field image (C). Scale bar is 20 μm.

**Labelling of extracellular antigens:** To investigate the specificity of commercial Qdot-Abs for intracellular targets, different types of proteins were stained simultaneously with conventional Dye-Abs and Qdot-Abs and imaged using an epifluorescence microscope. To eliminate the possibility of competition between the Dye-Abs and Qdot-Abs, for the antigen binding sites, which could affect the labelling, samples were also prepared separately with either a Dye-Ab or Qdot-Ab. A Qdot 625-Ab concentration of 20 nM was used, as it has been shown that a high concentration of Qdot-Abs improves specific labelling and signal-to-noise ratio [21]. Initially, to assess the labelling efficiency of Qdot-Abs, the extracellular matrix (ECM) protein fibronectin was dual labelled with Qdot 625-Ab and Dye-Ab (Alexa Fluor 488). Fibronectin is abundant at the cell surface and therefore, would not be expected to display any artifacts associated with accessibility. Indeed, in this case similar labelling was achieved with Qdot 625 and Alexa Fluor 488 (Figure 2).

**Figure 3:**
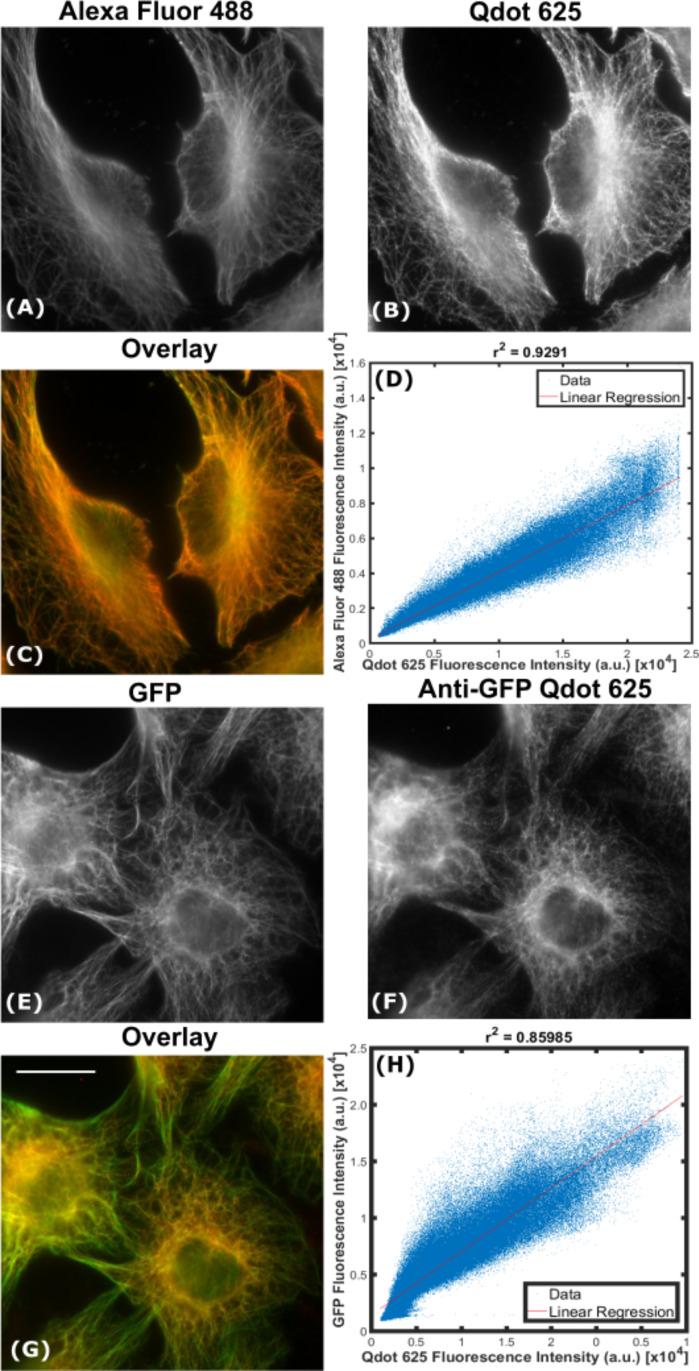
Specific labelling of tubulin with Qdots. Methanol-fixed HeLa cells were labelled indirectly with a primary anti-tubulin antibody, green Alexa Fluor 488 (A), and red Qdot 625 (B), to produce an overlaid wide-field image (C). As a measure of co-localisation between Alexa Fluor 488 and Qdot 625, fluorescence intensities of the overlaid wide-field image (C) were analysed using a custom-written Matlab code to produce a correlation scatter plot (D). Pearson’s correlation coefficient (*r*^2^) of 0.93 indicates very high correlation. The average Manders coefficient of Alexa Fluor 488 overlapping with Qdot 625 (M1) was 0.99 (SD = 0.007, *N* = 3) and the average Manders coefficient of Qdot 625 overlapping with Alexa Fluor 488 (M2) was 0.99 (SD = 0.005, *N* = 3, as determined using JACoP. Paraformaldehyde fixed TC7 cells, expressing tubulin-GFP (E), were also labelled directly with an anti-GFP Qdot 625 conjugate (F) to produce an overlaid wide-field image (G,) and a corresponding correlation scatter plot (H); with a Pearson’s correlation coefficient (*r*^2^) of 0.86. Scale bar is 20 μm.

**Labelling of cytosolic structures:** The vast majority of studies using Qdots show tubulin staining [6,12,16,18,20], therefore we sought to label this abundant cytosolic protein in order to have a positive control for our labelling protocol. After incubation with an anti-tubulin primary antibody and a Qdot 625-Ab, we once again found labelling which was comparable to samples (simultaneously or separately) labelled with Alexa Fluor 488 (Figure 3).

To investigate whether the same structure (microtubules) could be labelled with a different fluorophore-antibody conjugate, direct ICC was performed using a Qdot 625 conjugated directly to an anti-GFP primary antibody (see Methods for details). Cells expressing tubulin-GFP were labelled with the anti-GFP Qdot 625 conjugate (Qdot 625-GFP) and gave similar results to those imaged with indirect ICC (Figure 3). By inspection it was clear, that β-tubulin had been labelled with Qdot 625 (Figure 3C), however, to compare the two labelling techniques, an intensity correlation plot was produced (Figure 3D). A Pearson’s correlation coefficient (*r*^2^) value of 0.93 suggested that there was an almost perfect correlation between Qdot 625 and Alexa Fluor 488 labelled microtubules. In addition, the degree of co-localisation was measured using the Manders coefficient [29]. The average Manders coefficient of Alexa Fluor 488 overlapping with Qdot 625 (M1) was 0.99 (SD = 0.007, *N* = 3) and Qdot 625 overlapping with Alexa Fluor 488 (M2) was 0.99 (SD = 0.005, *N* = 3), confirming that the correlation between Qdot 625 and Alexa Fluor 488 was very good. In addition, an anti-tubulin primary antibody was also conjugated to Qdot 625 (Qdot 625-Tubulin) (Figure S3 in Supporting Information File 1). A negative control containing Alexa Fluor 488-Ab and Qdot 625-Ab only, with no anti-tubulin primary antibody was also prepared, showing negligible unspecific binding of Alexa Fluor 488 and Qdot 625 to HeLa cells (Figure S4 in Supporting Information File 1).

**Figure 4:**
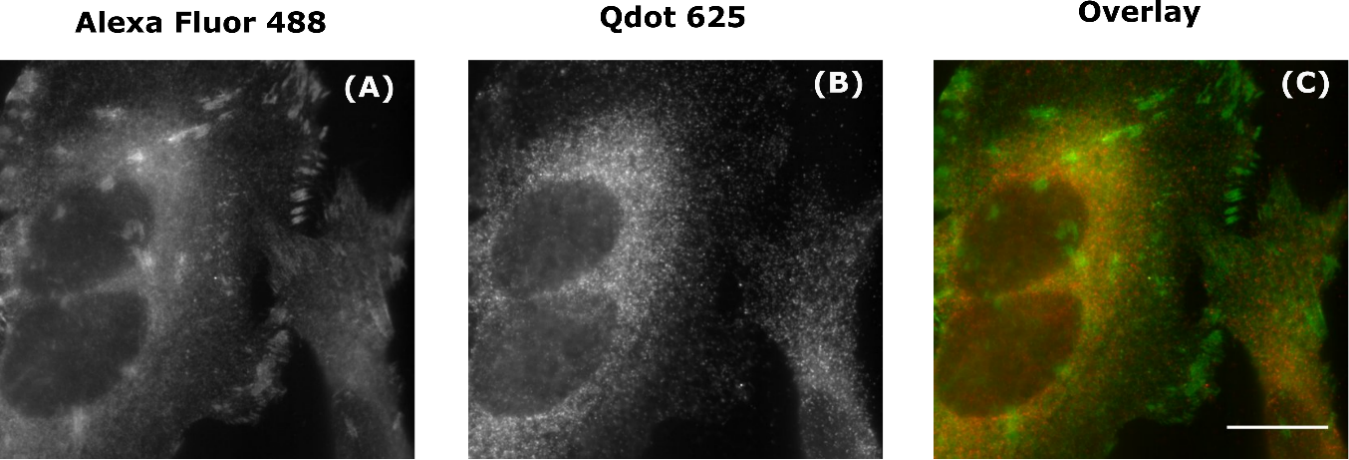
Non-specific labelling of talin with Qdots. Fixed HeLa cells were dual labelled with green Alexa Fluor 488 (A) and red Qdot 625 (B) to produce an overlaid wide-field image (C). Scale bar is 20 μm.

**Labelling intracellular complexes:** Both fibronectin and β-tubulin are highly abundant proteins. Furthermore, their antigens are relatively accessible. We therefore, looked to antigens present in more complex intracellular structures, including the focal adhesion protein talin and nuclear splicer marker SC35. Talin exists in a dynamic equilibrium with both a bound pool (forming focal adhesions) and a cytosolic pool. Using the same primary antibody, Alexa Fluor 488 labelled both the cytosolic pool of talin and the bound pool forming focal adhesions, whereas Qdot 625 appeared to only label the cytosolic regions (Figure 4). At the same time, a control sample was prepared, consisting of HeLa cells incubated simultaneously with an Alexa Fluor 488-Ab and Qdot 625-Ab, without prior addition of an anti-talin primary antibody (Figure S5 in Supporting Information File 1). Here, it appears that the low signal of Qdot 625, is an indication that that the Qdot 625-Ab actually binds non-specifically to cells.

**Figure 5:**
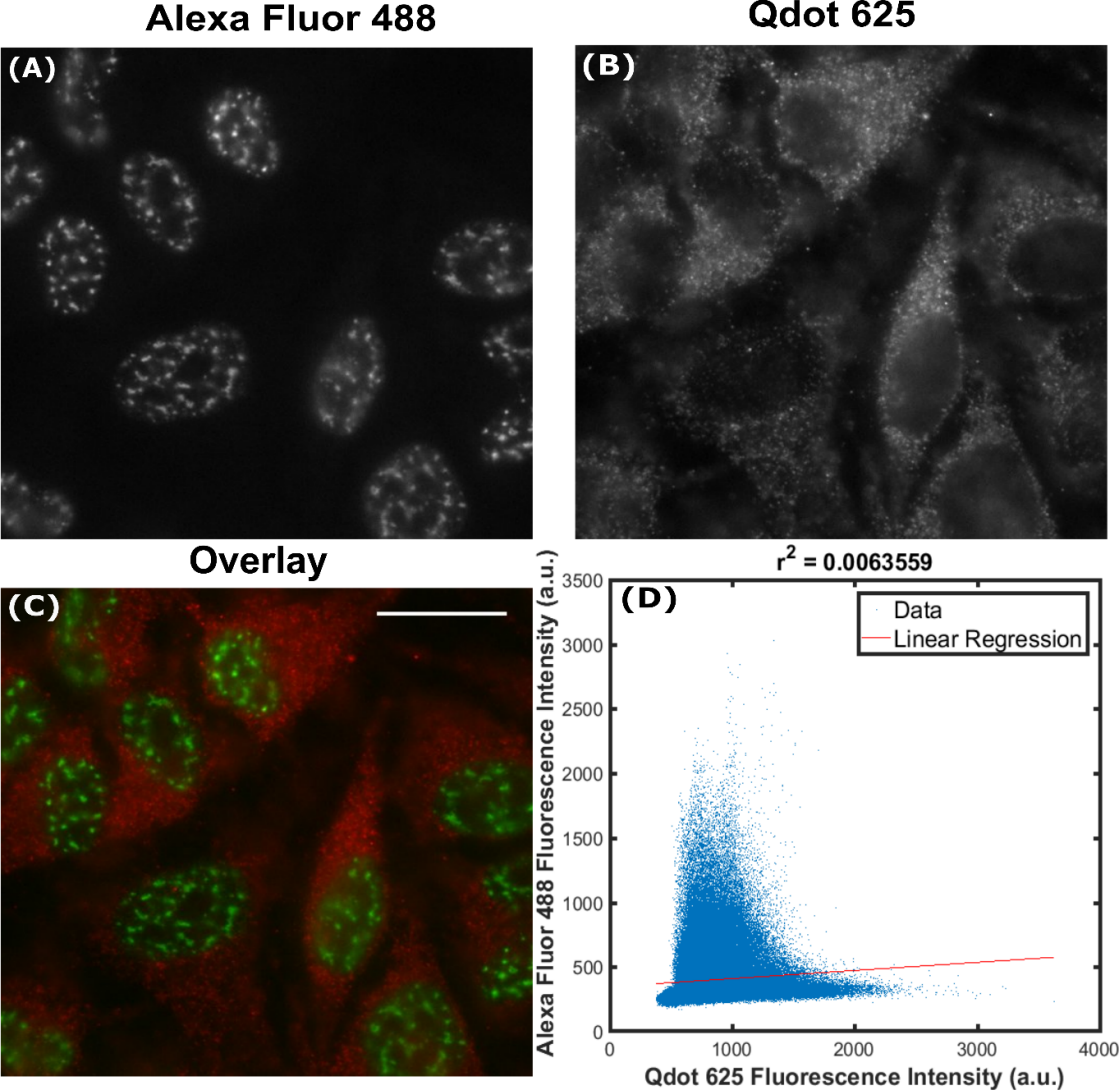
Non-specific labelling of SC35 with Qdots. Fixed HeLa cells were dual labelled with green Alexa Fluor 488 (A) and red Qdot 625 (B) to produce an overlaid wide-field image (C). Scale bar is 20 μm. As a measure of co-localisation between Alexa Fluor 488 and Qdot 625, fluorescence intensities of the overlaid wide-field image (C) were analysed using a custom-written Matlab code to produce a correlation scatter plot (D). Pearson’s correlation coefficient (*r*^2^) of 0.006 indicates no co-localisation. The average Manders' coefficient of Alexa Fluor 488 overlapping with Qdot 625 (M1) was 0.19 (SD = 0.035, *N* = 3) and the average Manders’ coefficient of Qdot 625 overlapping with Alexa Fluor 488 (M2) was 0.08 (SD = 0.031, *N* = 3), as determined using JACoP.

Unlike β-tubulin and talin, SC35 is not only intracellular, but contained within the nucleus, which is crowded with deoxyribonucleic acid (DNA) and proteins. Under conditions identical to the previous experiments, the labelling of SC35 with the Qdot 625-Ab was non-specific, diffuse, and predominately cytosolic (Figure 5). The intensity correlation of Qdot 625 and Alexa Fluor 488 labelling of SC35 was assessed by plotting fluorescence intensities (Figure 5D) within the overlaid image (Figure 5C). A Pearson’s correlation coefficient (*r*^2^) value of 0.006 suggested that there was practically no correlation between Qdot 625 and Alexa Fluor 488 signals. Once more, the degree of co-localisation was quantified using the Manders’ coefficient. The average Manders’ coefficient of Alexa Fluor 488 overlapping with Qdot 625 (M1) was 0.19 (SD = 0.035, *N* = 3) and the average Manders’ coefficient of Qdot 625 overlapping with Alexa Fluor 488 (M2) was 0.08 (SD = 0.031, *N* = 3), which confirms that the labelling of SC35 with Qdot 625 showed no co-localisation with Alexa Fluor 488. To control for non-specific binding of the Qdot 625-Ab to cells, a negative control was introduced, whereby the cells were incubated with the Qdot 625-Ab only (Figure S6 in Supporting Information File 1). In the absence of a primary antibody, there was negligible labelling detected with the Qdot 625-Ab, which suggests that the SC35 cytosolic signal is due to the presence of minority pool of SC35 in the cytosol.

**Optimising sample preparation:** Since Qdot-Abs may be sensitive to certain fixation protocols [13,19,30], as well as using paraformaldehyde, ice cold methanol was also tried. Methanol fixation and harsher permeabilisation with up to 1% Triton X-100 was used in an attempt to increase accessibility of the Qdots to complex intracellular antigens. Although methanol fixation gave better signal-to-noise ratio and specific Qdot 625 labelling of β-tubulin (Figure 3B), without the need for further permeabilisation, there was non-specific labelling observed for talin and SC35.

**Size limiting access to the nucleus:** We suspected that the size of the commercial Qdot 625-Ab and the associated accessibility to targets was the reason behind the inability to label complex cytosolic and nuclear structures. Although, the manufacturer (Thermo Fisher Scientific, UK) has approximated that Qdot 625 (17 nm) has an overall hydrodynamic size of 30 nm with a couple of antibodies per Qdot, we have provided our own measurements of Qdot 625 in the Supporting Information File 1. We attempted to measure the size of Qdot 625 using different methods: transmission electron microscopy (TEM), size-exclusion high-performance liquid chromatography (SEC-HPLC), and fluorescence correlation spectroscopy (FCS). The TEM and SEC-HPLC results are in agreement with the provider’s data, giving a core size of ≈8 nm (Figure S7 in Supporting Information File 1) and a hydrodynamic diameter of ≈15 nm (Figure S8 in Supporting Information File 1), respectively. However, the FCS results indicate a much larger size of ≈76.84 nm for the Qdot 625-Ab (Figure S9 in Supporting Information File 1), suggesting some degree of Qdot aggregation (although this is not seen in the SEC-HPLC nor in the TEM data). Due to the discrepancy observed for Qdot 625-Ab, using different methods, we decided to take FCS measurements for a presumably smaller green Qdot 525-Ab. The size obtained for the Qdot 525-Ab was more compatible with the provider’s data at 41.72 nm (Figure S10 in Supporting Information File 1). ATTO488 (Figure S11 in Supporting Information File 1) was used to calibrate the confocal volume and fluorescent latex beads (Figure S12 in Supporting Information File 1), of a known size, were used as a standard to compare against Qdot-Abs (For methods see Supporting Information File 1). These results are robust and reproducible (including two different batches of Qdot 625-Ab).

**Figure 6:**
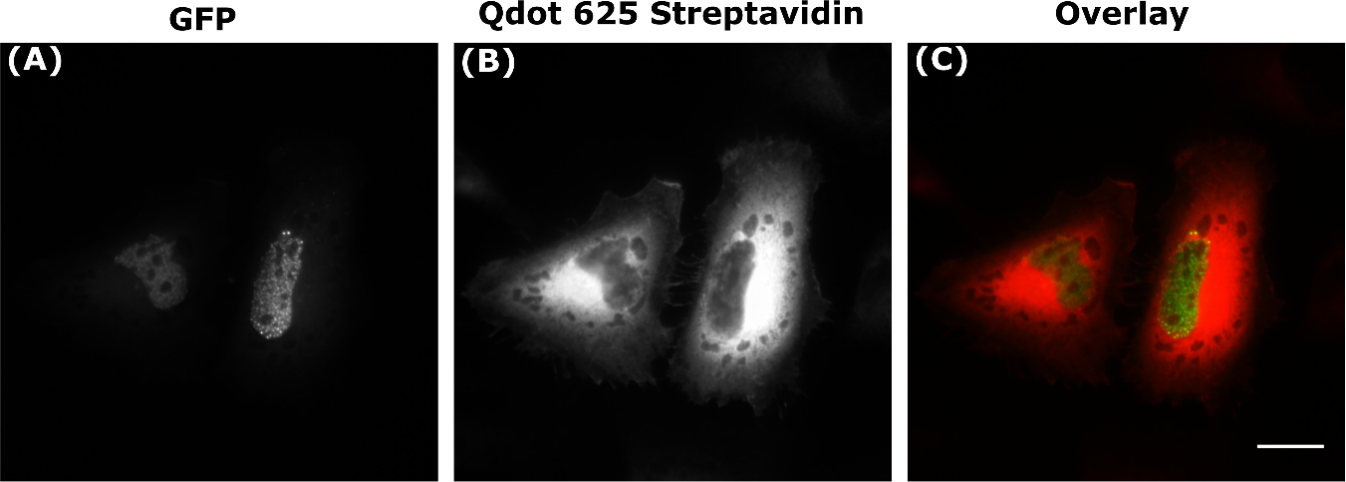
Partial labelling of HIF2α with Qdot-Streptavidin. Fixed HeLa cells were transfected with EGFP-HIF2α (A), incubated with a primary anti-GFP biotinylated antibody and Qdot 625 streptavidin conjugate (Qdot-Streptavidin) (B), to produce an overlaid image (C). Scale bar is 20 µm.

**Labelling with alternative Qdot-Abs:** A Qdot 525 conjugated secondary antibody (Qdot 525-Ab), with presumably smaller dimensions, was further tried against the same anti-tubulin primary antibody (Figure S13 in Supporting Information File 1), anti-talin primary antibody (Figure S14 in Supporting Information File 1), and anti-SC35 primary antibody (Figure S15 in Supporting Information File 1), with the similar results obtained as for Qdot 625-Ab. An alternative Qdot 625 conjugated to the biotin-binding protein streptavidin (Qdot-Streptavidin) (Thermo Fisher Scientific, UK) was also evaluated. To test the specificity of Qdot-Streptavidin for nuclear targets, a transcription factor, which localises in the nucleus as speckles (sub-nuclear foci), known as hypoxia inducible factor two alpha (HIF2α) was labelled. Fixed HeLa cells were transfected with HIF2α tagged with the fusion protein EGFP (EGFP-HIF2α), incubated with anti-GFP biotin primary antibody, and Qdot-Streptavidin. All of the endogenous biotin sites in the cell were blocked before addition of the biotinylated anti-GFP primary antibody with an endogenous biotin-blocking kit (Thermo Fisher Scientific, UK). Similar results were obtained as previously for Qdot 625-Ab, with Qdot-streptavidin binding to any cytosolic pool of HIF2α without labelling the distinct speckles in the nucleus (Figure 6). Since the transfected cells have a cytosolic pool of EGFP-HIF2α, this was labelled by Qdot-streptavidin more than in the untransfected cells, where there was no GFP present; hence the very bright fluorescent Qdot signal in the cytosol.

**Figure 7:**
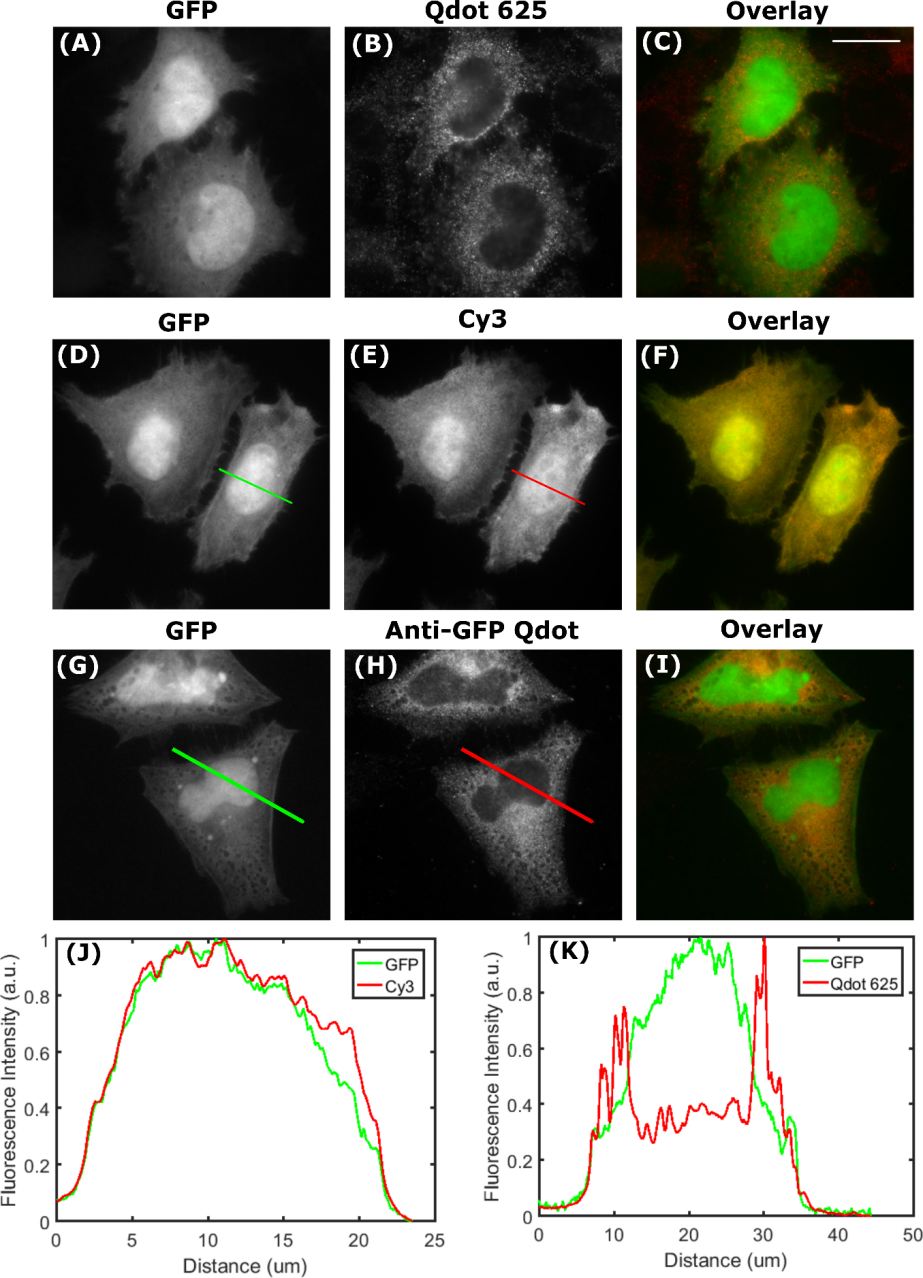
Qdot-Abs are unable to access the cell nucleus. Fixed HeLa cells were transfected with unconjugated soluble GFP (A), incubated with a primary anti-GFP antibody, and red Qdot-Ab (B) to produce an overlaid image (C). Fixed HeLa cells were also transfected with unconjugated soluble GFP (D), incubated with a primary anti-GFP antibody, and red cyanine 3 conjugated to a secondary antibody (E) to produce an overlaid image (F). Direct ICC was done by incubating fixed HeLa cells, transfected with unconjugated soluble GFP (G), with an anti-GFP Qdot 625 conjugate (H) to produce an overlaid image (I). Normalised fluorescence intensities of unconjugated soluble GFP labelled with Cy3 (J) and Qdot 625 (K) were plotted from corresponding lines scans to show no labelling within the nucleus with Qdot 625. Scale bar is 20 μm.

**Assessing nuclear accessibility of Qdots:** Fluorescent in situ hybridisation (FISH) is another application where specific intracellular targeting is required. Like many applications, FISH benefits from bright photostable probes, and indeed several studies have been published using Qdots for FISH [31]. Much of the FISH data however, describe metaphase chromosomes (where breakdown of the nuclear envelope has occurred) or chromosomal spreads devoid of cell membranes altogether [32–34]. Furthermore a 2009 appraisal of the use of Qdots for FISH considers them unsuitable in their current form "… because of the lack of reproducibility of the experiments" when using these materials [35]. Interestingly, in all of our Qdot 625 images, even when the staining was non-specific, there was no labelling within the nucleus (Figure 2–6). This suggested that, specificity issues aside, the Qdot-Abs could not access the nucleus at all. This hypothesis was tested by transfecting cells with an unconjugated soluble GFP, which diffuses throughout the cytoplasm and nucleus. We then labelled with either a direct anti-GFP Qdot 625 conjugate (Qdot-GFP) or indirectly with a primary anti-GFP antibody and Qdot 625-Ab. Both approaches yielded homogenous labelling in the cytosol, with the Qdot signal being excluded from the nucleus (Figure 7). There was also little non-specific Qdot 625 staining in the non-transfected cells (Figures S16 and S17 in Supporting Information File 1). Unlike Qdot 625, when the unconjugated soluble GFP was immunolabelled with a secondary antibody coupled to the fluorescent dye cyanine 3 (Cy3), there was labelling in both the cytosol and nucleus (Figure 7E). To assess the extent at which Qdot 625 did not label soluble GFP within the nucleus, fluorescence intensities of Qdot 625 and GFP were plotted from line scans taken across a section of the cell, including the cytosolic region and nucleus (Figure 7K). There was an obvious decline in the fluorescence intensity of Qdot 625 in the nucleus. Under continuous illumination, the fluorescence intensity of Qdot 625 in the cytosol was greater than that of GFP, due to the superior brightness of Qdots. Multichannel images were taken without adjustment to the focus to rule out different focal planes explaining the absence of Qdots from the nucleus.

## Conclusion

Fluorescent Qdots are bright and photostable and hence have been promoted as having significant potential for imaging in biology, including for the immunolabelling of cellular structures. However, even after several years of promising articles and reviews, Qdots have not found routine use in biological research. Here, we examine the performance of some of the few Qdot conjugates commercially available for immunofluorescence (Table S1 in Supporting Information File 1). While all tested antigens could be labelled using antibodies conjugated to fluorescent dyes (Dye-Abs) (e.g. Alexa Fluor 488), only tubulin and extracellular fibronectin could be labelled specifically with Qdot-Abs. Neither a nuclear protein (SC35) nor an antigen within a large complex (talin) could be labelled with the Qdot-Abs. Our hypothesis is that the specificity of Qdot-Abs is dependent upon the type of protein, its abundance, and location within the cell. This is most likely due to the size of the Qdot-Abs, leading to steric hindrance and limited access to certain epitopes. Additionally, if cross-linking of the target protein has occurred, Qdot 625 aggregates may restrict access to the epitope and thus affect the specific labelling of these proteins [36].

Therefore, we conclude that these Qdot-Abs are not suitable to detect complex intracellular structures unless the proteins are abundant and have multiple, accessible antigens along the structure. Although Montón et al. suggest that Qdot-Abs were more specific for proteins that are scarce in the cell [20], here we find that the specific labelling of proteins such as SC35 and talin could not be achieved with the with commercial Qdot-Abs.

The parameters that may affect the ability of Qdots to penetrate intracellular targets (such as foci adhesion complexes or nuclear proteins) include the labelling ratio between Qdots and functional antibodies, the overall surface charge of Qdot-Ab conjugates, and whether there is a presence of a protein corona. The labelling ratio of Qdots to antibodies present within commercially available Qdot-Ab conjugates has been previously evaluated, where the non-specific labelling of proteins was attributed to the lack of IgG molecules per Qdot [37]. Depending on the chosen Qdot-Ab conjugation method, the binding affinity of the antibody for the Qdots can be increased. However, regardless of the variability in the Qdot synthesis and antibody conjugation protocols, the orientation in which the IgG molecules bind to Qdots cannot be controlled [37]. The binding affinity of Qdot-Abs can also be affected by the surface charge they possess. For instance, positively charged Qdots are taken up by cells more readily than negatively charged or zwitterionic Qdots, which suppress protein adsorption and thus the formation of a protein corona [38]. Qdots with a zwitterionic surface have a zeta potential of near zero, are resistant to non-specific binding onto cells, and have a high colloidal stability [39]. Commercially available Qdot-Abs evaluated in this report were used in fixed, permeabilised cells that were blocked with BSA (negatively charged). One way to assess the non-specific binding of these Qdot-Abs to cells would be to use flow cytometry.

Beyond those commercially available Qdots, there have been a number of ICC protocols published for the labelling of different proteins with Qdot-Abs [6,20,24]. These reports, however, were mostly focused on the unproblematic labelling of tubulin for their proof-of-principle experiments. We suggest that future developments of Qdot-Abs include other more challenging targets as benchmarks, for example, those evaluated in this article. The ideal scenario would be a toolbox of commercially available Qdot-Abs that can be consistently used to label any biological structure of interest and not just tubulin and extracellular targets.

## Methods

### Cell culture

Human cervix epithelioid carcinoma (HeLa, ECACC number 930210a3) cells were cultured in a 75 cm^2^ flask at 37 °C with 5% CO_2_, minimum essential media (MEM, Life Technologies, UK) supplemented with 10% (v/v) foetal calf serum (FCS), and 1% non-essential amino acids (NEAA). Cells were split 1,000,000 cells/mL when ≥80% confluent with trypsin-EDTA. Rat mammary (Rama) 27 fibroblasts were cultured in a 75 cm^2^ flask at 37 °C with 5% CO_2_, Dulbecco's modified Eagle's medium (DMEM, Life Technologies, UK) supplemented with 10% (v/v) FCS (Life Technologies, UK), 0.75% (w/v) sodium bicarbonate, 4 mM L-glutamine, 50 ng/mL insulin, and 50 ng/mL hydrocortisone (Sigma-Aldrich, UK), as described previously [40]. Cells were split 1:8 when ≥60% confluent with trypsin-EDTA. A stable cell line TC7 3xGFP (expressing tubulin-GFP) was cultured in a 75 cm^2^ flask at 37 °C with 5% CO_2_, MEM (Life Technologies, UK) supplemented with 10% (v/v) FCS, 1% NEAA, and genetitin (Sigma-Aldrich, UK), as described previously [41]. Cells were split 1:15 when ≥80% confluent with trypsin-EDTA.

### Transfection

HeLa cells were seeded onto 16 mm glass coverslips (100,000 cells/mL) in a 12-well plate and transfected with pG-EGFP-A (soluble GFP) or pG-EGFP-HIF2α (EGFP-HIF2α) using FuGENE6 transfection reagent (Roche Limited, UK), following the manufacturer's protocol (3:1 transfection reagent/DNA plasmid).

### Site click conjugation of Qdot625 to anti-GFP

Following the manufacturer's protocol, a commercial site-click Qdot 625 antibody conjugation kit (Thermo Fisher Scientific, UK) was used to conjugate a primary mouse (clones 7.1 and 13.1) anti-GFP antibody (Roche Limited, UK) to dibenzocyclooctyne (DIBO) modified Qdot 625. The concentration of the Qdots in the conjugate was calculated to be 3 μM using the equation *c* = *A*/ε, where *c* is the concentration of DIBO-modified Qdot 625 attached to the primary antibody, *A* is the absorbance of Qdot 625, and ε is the extinction coefficient of Qdot 625 (500,000 M^−1^·cm^−1^). The absorbance between 605–612 nm (step 10 nm) was measured to be 1.5 a.u. using a quartz cuvette with a 1 cm path length, on a SpectraMax 34 Plus spectrophotometer (Molecular Devices, UK).

### Immunofluorescence

**Figure 8:**
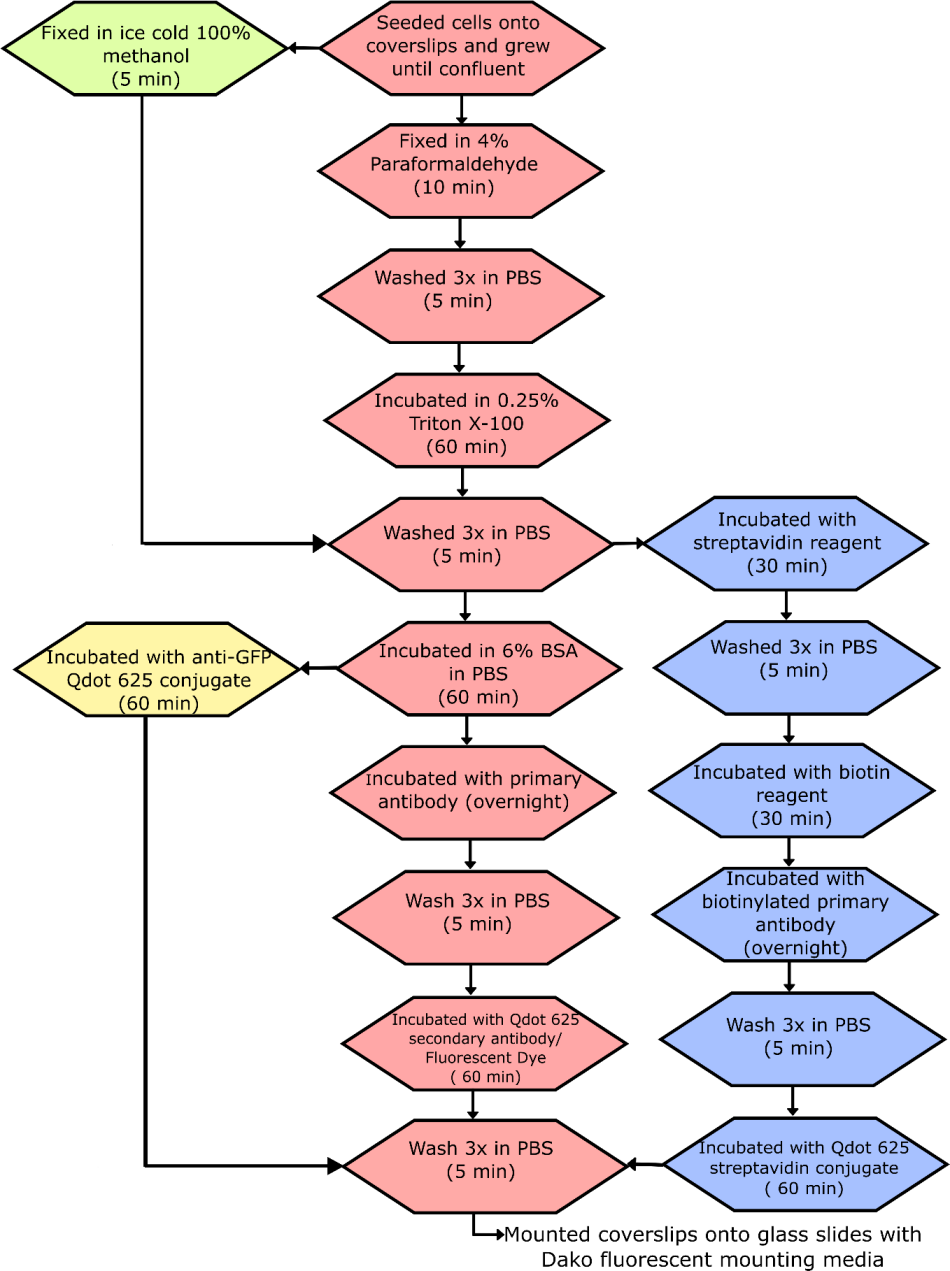
Immunofluorescence protocol. The pink boxes show the method for use with a primary antibody and Qdot 625/Fluorescent dye conjugated to a secondary antibody, green box is for methanol fixation, yellow boxes for the anti-GFP Qdot 625 conjugate, and blue boxes are for biotinylated primary antibody with Qdot 625 streptavidin conjugate.

An overview of the immunofluorescence procedure is shown in Figure 8. Briefly, cells were seeded onto glass coverslips and grown until confluent, washed once in phosphate buffered saline (PBS) (37 °C), and fixed in 4% (w/v) paraformaldehyde (PFA) for 10 min or 100% ice cold methanol (5 min). Cells were washed 3× in PBS (5 min), permeabilised with 0.25% Triton X-100 in PBS for 60 min (except methanol fixation), washed again 3× in PBS (5 min), and incubated with 6% bovine serum albumin (BSA, Sigma-Aldrich, UK) in PBS (60 min). Primary antibodies produced in mouse (anti-β-tubulin TUB 2.1, Sigma-Aldrich, UK; anti-GFP Roche Limited, UK; anti-SC35, Abcam, UK; and anti-talin, Sigma-Aldrich, UK), those produced in rabbit (anti-fibronectin, Sigma-Aldrich, UK), and biotinylated anti-GFP (Abcam, UK) were diluted 1:100 in 6% BSA and incubated overnight at 4 °C. Cells were washed 3× in PBS (5 min) and incubated simultaneously with either a mixture of Donkey anti-mouse IgG H+L secondary antibody Qdot 625 conjugate, Qdot 625 anti-GFP conjugate, F(ab')2-Goat anti-rabbit IgG H+L secondary antibody Qdot 625 conjugate, or Qdot 625 streptavidin conjugate (Thermo Fisher Scientific, UK), diluted 1:50 to 20 nM in 6% BSA, and goat anti-rabbit Alexa Fluor 488 or goat anti-mouse Alexa Fluor 488 (Thermo Fisher Scientific, UK), diluted 1:500 to 4 µg/mL in 6% BSA; or anti-mouse cyanine 3 (Sigma-Aldrich, UK) diluted 1:500 to 4 µg/mL in 6% BSA, at room temperature (60 min). Before preparation of the Qdot 625 conjugated secondary antibody, the vial was centrifuged at 5,000 g for 3 min to remove any aggregates. After 3 washes in PBS (10 min), coverslips were mounted onto slides with Dako fluorescent mounting media (Dako, UK), and stored at 4 °C. A negative control of Qdot 625 conjugated antibody only was also prepared to show any background staining.

### Wide-field imaging

Images were taken on a wide-field epifluorescence microscope (Carl Zeiss Axio Observer Z.1, Germany) with a 16 μm 512 × 512 pixel sensitive electron-multiplying charge-coupled device (EMCCD) camera (Andor iXon 897 Ultra), polychromatic mercury arc lamp, 39106-AT-QDot 625 filter set (Chroma Technology Corporation, USA), and a 100× 1.45 NA oil-immersion objective. Fluorescent and corresponding brightfield images were acquired using Micro-Manager software [42]. The same acquisition settings were used for each set of images, including lamp power, exposure time, and gain.

### Co-localisation analysis

Pearson correlation coefficient scatter plots of Qdot 625 and Alexa Fluor 488/GFP were produced using a custom-made Matlab code [43]. Manders’ correlation coefficients were determined using a Just Another Co-localization Plugin (JACoP) [29] in FIJI [44].

## Supporting Information

File 1 Additional data.
