# Supplementary material for: Evaluation of quantum dot conjugated antibodies for immunofluorescent labelling of cellular targets
Source: Beilstein J Nanotechnol. 2017 Jun 9;8:1238–49. doi: 10.3762/bjnano.8.125 (PMC5480344; doi:10.3762/bjnano.8.125)
Supplement: File 1 — Additional data. [file Beilstein_J_Nanotechnol-08-1238-s001.pdf]

Supporting Information
for
Evaluation of quantum dot conjugated antibodies for immunofluorescent labelling of cellular targets

Jennifer E. Francis[1], David Mason[2], and Raphaël Lévy[*1]

Address: [1]Department of Biochemistry, Institute of Integrative Biology, Biosciences Building, Crown Street, Liverpool, L69 7ZB, United Kingdom and [2]Centre for Cell Imaging, Institute of Integrative Biology, Biosciences Building, Crown Street, Liverpool, L69 7ZB, United Kingdom

Email: Raphaël Lévy - rapha@liverpool.ac.uk

*Corresponding author

Additional Data

Introduction

In this Supporting Information, we provide:

1.1 The emission and excitation spectra for Alexa Fluor 488, Qdot 625, Cy3, and Qdot 525

2.1 Results from the conjugation of anti-tubulin primary antibody and Qdot 625

3.1 Negative controls with Qdot 625-Ab

4.1 Size measurements: TEM, SEC-HPLC, and FCS

5.1 Labelling with Qdot 525-Ab

6.1 Non-transfected controls

7.1 Qdot labelling summary table

1.1 Emission and excitation spectra

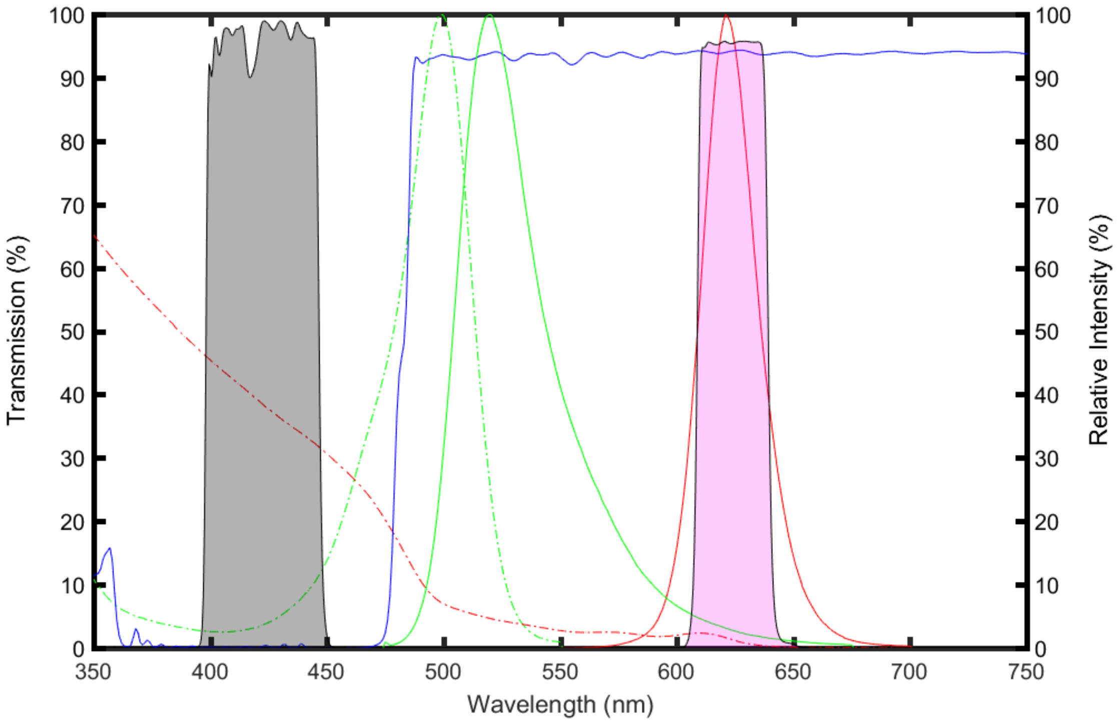

Figure S1: Excitation and emission spectra of Qdot 625-Ab and Alexa Fluor 488-Ab. Excitation of Qdot 625-Ab (red dotted line) and Alexa Fluor 488-Ab (green dotted line) were plotted along with the emission of Qdot 625-Ab (red solid line) and Alexa 488-Ab (green solid line). Data for the excitation and emission spectra was exported from Fluorescence SpectraViewer on the Thermo Fisher Scientific, UK website. A Qdot 625 filter set (39106, Chroma, UK) has also been added to the spectra, with excitation (grey shaded region), beam splitter (solid blue line), and emission (red shaded region), which were obtained from the Chroma website.

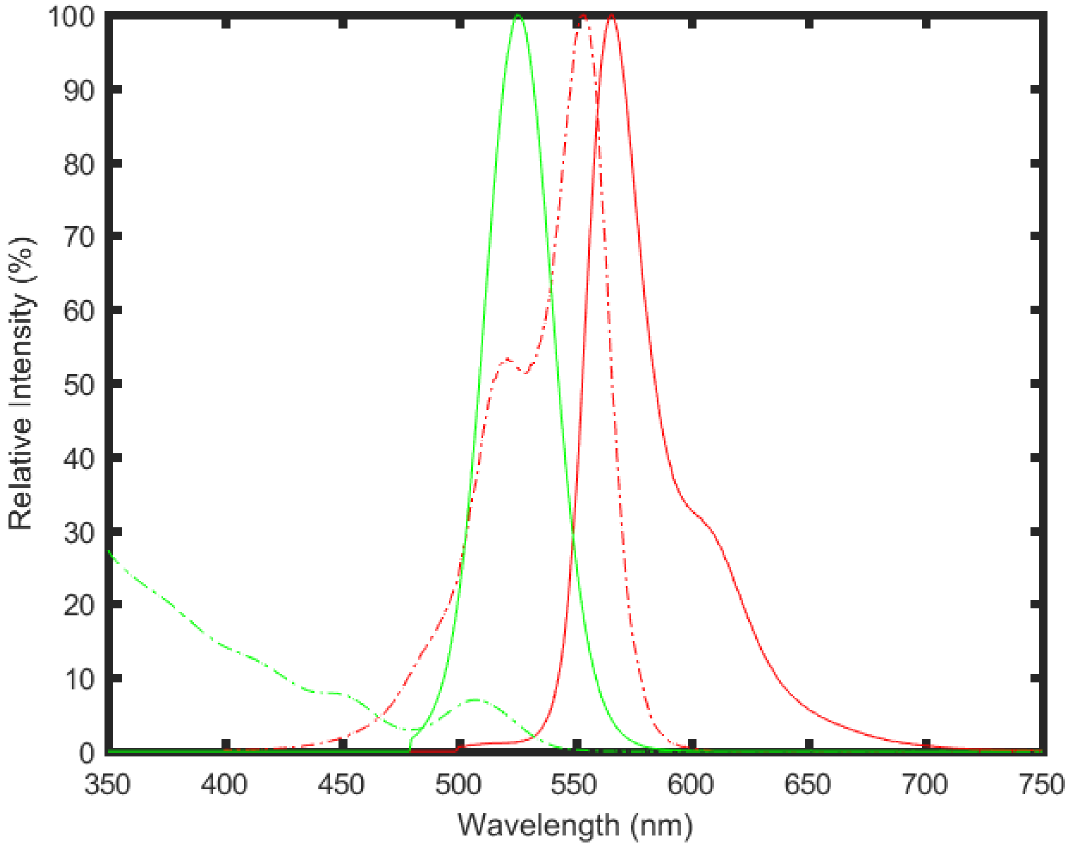

Figure S2: Excitation and emission spectra of Qdot 525-Ab and Cy3-Ab. Excitation of Qdot 525-Ab (green dotted line) and Cy3-Ab (red dotted line) were plotted along with the emission of Qdot 525-Ab (green solid line) and Cy3-Ab (red solid line). Data was exported from Fluorescence SpectraViewer on the Thermo Fisher Scientific, UK website.

2.1 Anti-tubulin Qdot 625 conjugate

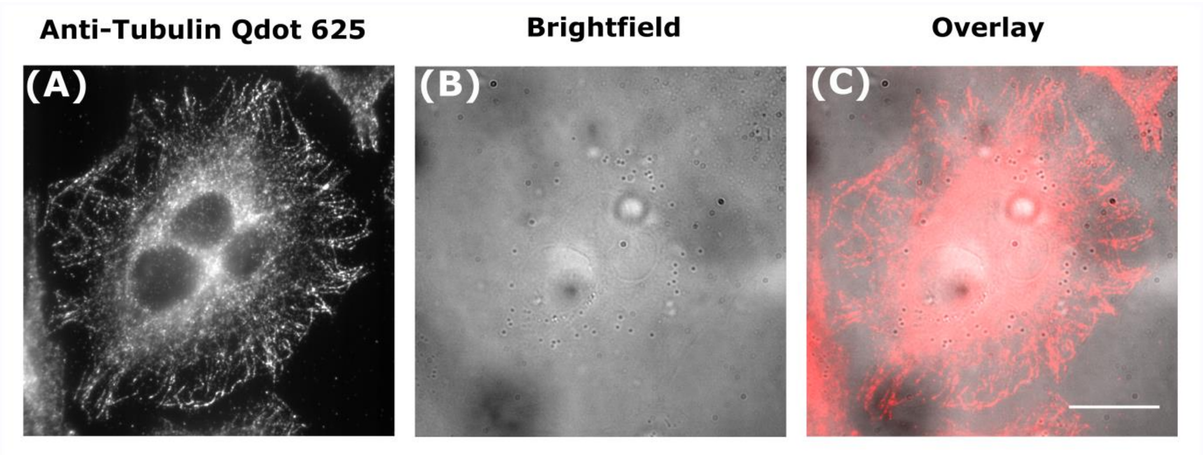

Figure S3: Specific labelling of tubulin with anti-tubulin Qdot 625 conjugate. An anti-tubulin primary antibody was conjugated to Qdot 625 via click chemistry (as described in the

Methods section of the paper for anti-GFP). HeLa cells were fixed and incubated with an anti-tubulin Qdot 625 conjugate (A). A corresponding brightfield image was taken (B) and overlaid image produced (C). Scale bar is 20 µm.

3.1 Negative controls with Qdot 625-Ab

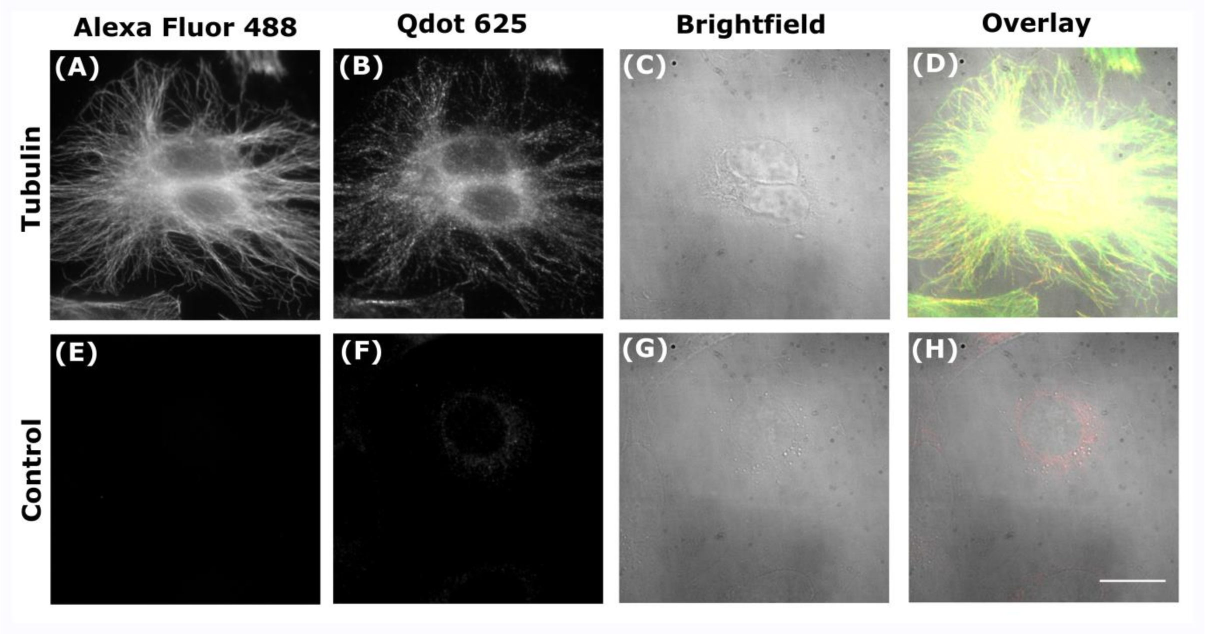

Figure S4: Labelling of Tubulin with Qdot 625. Fixed HeLa cells were dual labelled with green Alexa Fluor 488 (A) and red Qdot 625 (B). A corresponding brightfield image of HeLa cells (C) was also taken to produce an overlaid wide-field image (D). A negative control of HeLa cells incubated simultaneously with green Alexa Fluor 488 (E) and red Qdot 625 (F), without the addition of a primary antibody, was done. The corresponding brightfield (G) and overlay image (H) is shown. Scale bar is 20 µm.

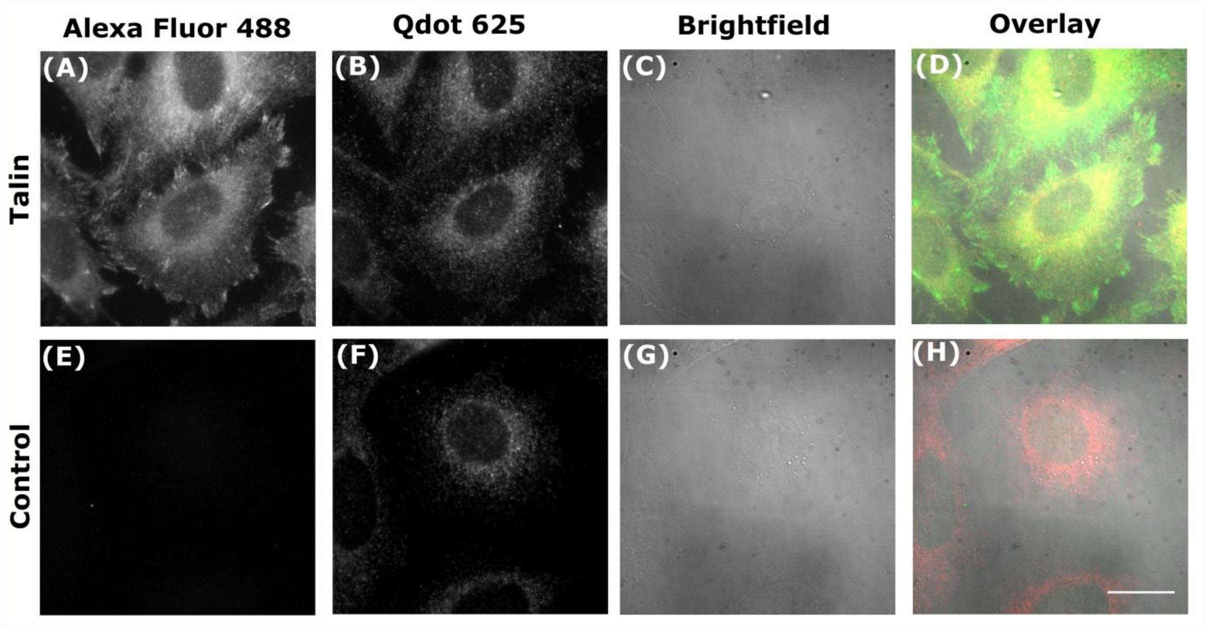

Figure S5: Labelling of Talin with Qdot 625. Fixed HeLa cells were dual labelled with green Alexa Fluor 488 (A) and red Qdot 625 (B). A corresponding brightfield image of HeLa cells (C) was also taken to produce an overlaid wide-field image (D). A negative control of HeLa cells incubated simultaneously with green Alexa Fluor 488 (E) and red Qdot 625 (F), without the addition of a primary antibody, was done. The corresponding brightfield (G) and overlay image (H) is shown. Scale bar is 20 µm.

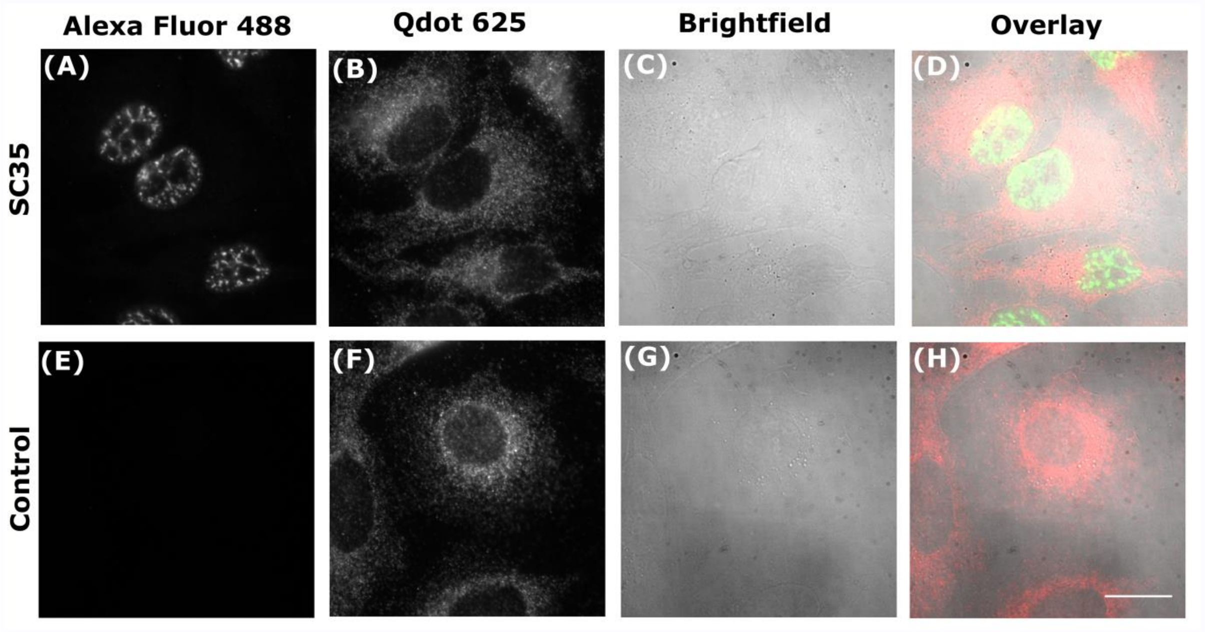

Figure S6: Labelling of SC35 with Qdot 625. Fixed HeLa cells were dual labelled with green Alexa Fluor 488 (A) and red Qdot 625 (B). A corresponding brightfield image of HeLa cells (C) was also taken to produce an overlaid wide-field image (D). A negative control of HeLa cells incubated simultaneously with green Alexa Fluor 488 (E) and red Qdot 625 (F), without the addition of a

primary antibody, was done. The corresponding brightfield (G) and overlay image (H) is shown. Scale bar is 20 µm.

4.1 Size measurements: TEM, SEC-HPLC, and FCS

Transmission electron microscopy method:

Qdot 625-Ab was centrifuged at 1,000 g for 5 min to remove any aggregates. A 5 µL sample (0.5 µL Qdot 625-Ab 1 µM, diluted in 100 µL water) was added to a formyar/carbon electron microscopy grid and left to air dry before imaging. Transmission electron micrograph image of Qdot 625 core/shell was taken on a Tecnai G3 spirit transmission electron microscope (TEM). The core/shell size of Qdot 625 was measured for all particles in the calibrated TEM image using the Analyze particles tool in Fiji and a bar chart plotted in Matlab to show the distribution of core/shell sizes. A threshold was set and joint particles were separated by applying a watershed filter.

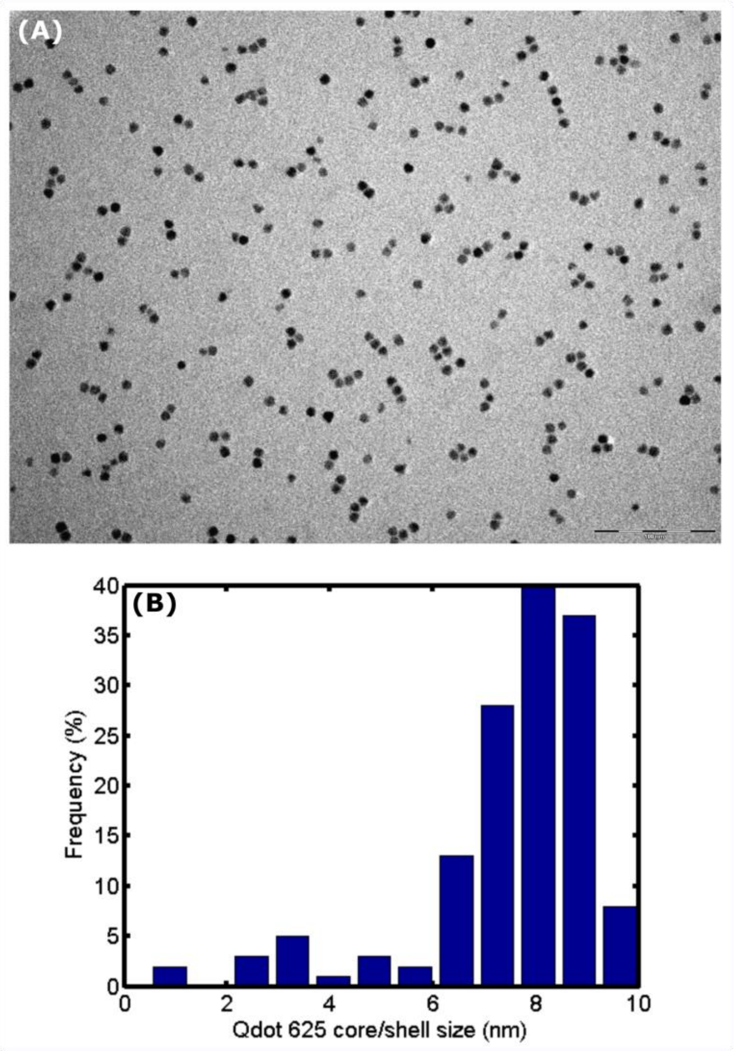

Figure S7. TEM of Qdot 625-Ab. Core/shell of Qdot 625 was imaged using TEM (A) and corresponding diameters plotted as a bar chart to determine the average core/shell size of Qdot 625 to be 8 nm (B). Scale bar is 100 nm.

Size-exclusion-high performance liquid chromatography (SEC-HPLC) method:

A BIO-Gel TSK 40 XL [300 x 7.8 mm] size-exclusion chromatography (SEC) column [Bio-Rad] was set up on a Dionex ICS-3000 high-performance liquid chromatography (HPLC) system and pre-equilibrated with 0.2 um filtered PBS. A 50 µL Qdot 625-Ab (0.1 µM) sample was injected into the column and run with PBS at a flow rate of 0.5 mL/min. Elution was monitored at 214, 280, 400, 500 and 600 nm. The column was calibrated by running standard proteins under the same conditions.
The SEC-HPLC result for Qdot 625-Ab, using the 214 nm detection wavelength, was plotted. Standard proteins of a known molecular weight were used as markers to calculate the molecular weight of Qdot 625-Ab.

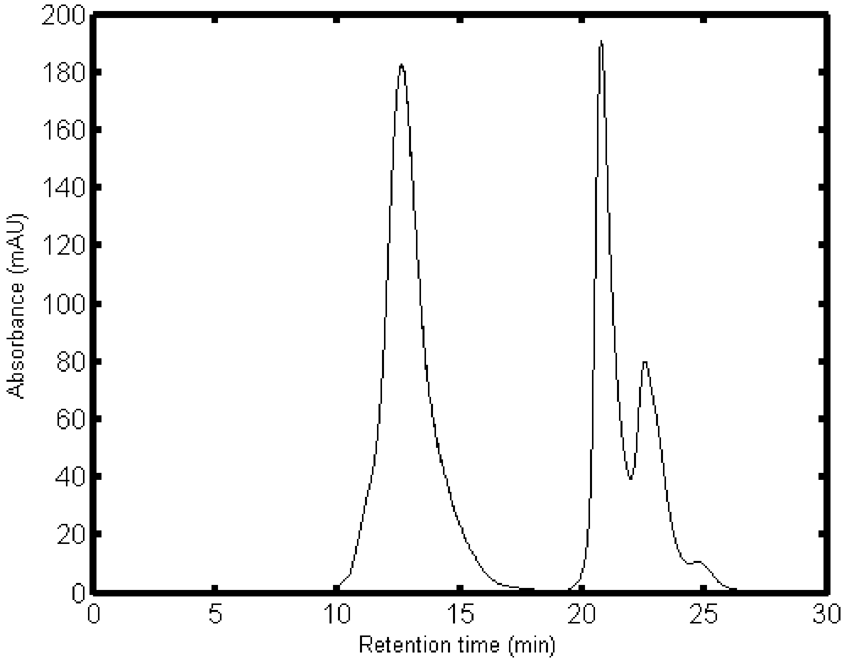

Figure S8. SEC-HPLC chromatograph for Qdot625-Ab. Molecular weight of Qdot625-Ab was determined to be 1,100 kDa (~15 nm) by comparing against known standard proteins. Using PBS as a buffer, Qdot625-Ab was eluted at 13 min using a 214 nm detection wavelength.

Fluorescence correlation spectroscopy (FCS) method:

A Zeiss Axio Observer Z.1 780 confocal microscope (Zeiss, Germany) attached to a PicoQuant system (PicoQuant GmbH, Germany) was used. Point measurements were acquired for 60 s at 37 °C using a 40x 1.2 NA water-immersion objective and a 485 nm pulsed diode laser (POL 828 Sepia II Multichannel Picosecond Diode Laser, PicoQuant GmbH, Germany). Samples were loaded into a custom-built chamber made out of a glass coverslip and Parafilm welded onto a glass microscope slide using a heating block to prevent evaporation. The sample was drawn up into the channel by capillary action and inverted with the glass coverslip facing the objective. For calibration of the

confocal volume (r_0), 10 nM ATTO 488 (ATTO-Tec, Germany) was used. Since the diffusion coefficient (D) of 400 µm²/s for ATTO 488 was known at a temperature (T) of 25 °C (PicoQuant, Germany), the diffusion coefficient (D) of ATTO 488 was determined to be 536 µm²/s at 37 °C using D (T) = D (25 °C) x $\frac{T\ (K)}{298.15\ K}$ x $\frac{(8.9x\ 10^{-4}\ PaS)}{n(T)}$, where n is the viscosity of water. During calibration, the correction collar of the 40x water-immersion objective was adjusted to 0.17 mm to match the thickness of the coverslip used. ATTO 488 was fitted with a pure diffusion model and r_0 was determined to be 0.6.fL after fixing the concentration to 10 mM and D to 536 µm²/s. A standard reference sample of 30 nm fluorescent latex beads (Sigma-Aldrich, UK), diluted 1:500 in water, was used as a comparison to the Qdot-Abs. Each FCS measurement was repeated 5 times at different positions in the channel and the 5 repeats for 1 position plotted. A 615 nm long pass filter was placed in front of the detector for Qdots to block excitation light and a 520/35 short band pass filter was used for the ATTO 488 and fluorescent beads. SymPhotime software (PicoQuant, Germany) was used to fit the autocorrelation function of ATTO488/beads to a pure diffusion model and the Qdot-Abs to a triplet model. The hydrodynamic diameter of the Qdot625-Ab and Qdot525-Ab was determined using: $d_{Qot} = d_{ST}$ x $\frac{\tau_{Qdot}}{\tau_{ST}}$, where d is the hydrodynamic diameter, Qdot refers to quantum dot, ST is the standard, and τ is the diffusion time [1].

Fluorescence correlation spectroscopy (FCS) results:

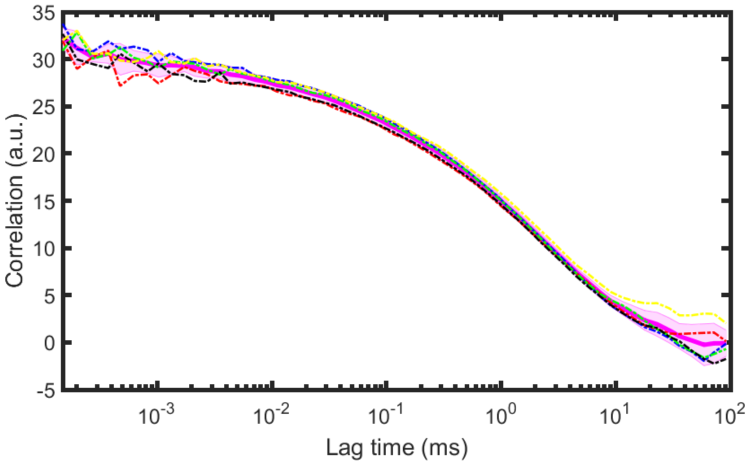

Figure S9. Fluorescence autocorrelation curves of Qdot 625-Ab. FCS measurements were taken *of Qdot 625-Ab (2 nM) diffusing in a confocal volume. The blue dotted line is repeat 1, red dotted line is repeat 2, green dotted line is repeat 3, black dotted line is repeat 4, and yellow dotted line is repeat 5. The solid pink line is the mean, with the shaded pink region showing the standard error. The hydrodynamic size of Qdot625-Ab, with an average diffusion time of 2.3 ms (N=5), was calculated to be 76.84 nm.*

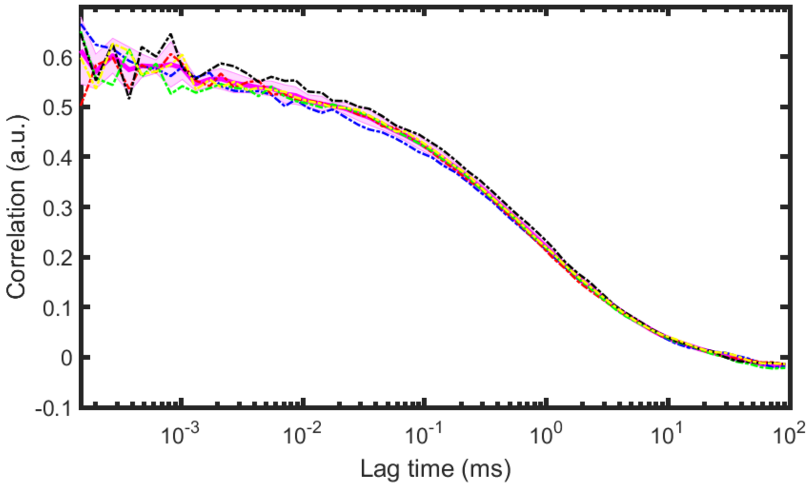

Figure S10. Fluorescence autocorrelation curves of Qdot 525-Ab. FCS measurements were taken *of Qdot 525-Ab (2 nM) diffusing in a confocal volume. The blue dotted line is repeat 1, red dotted line is repeat 2, green dotted line is repeat 3, black dotted line is repeat 4, and yellow dotted line is repeat 5. The solid pink line is the mean, with the shaded pink region showing the standard error. The hydrodynamic size of Qdot525-Ab, with an average diffusion time of 1.25 ms (N=5), was calculated to be 41.72 nm.*

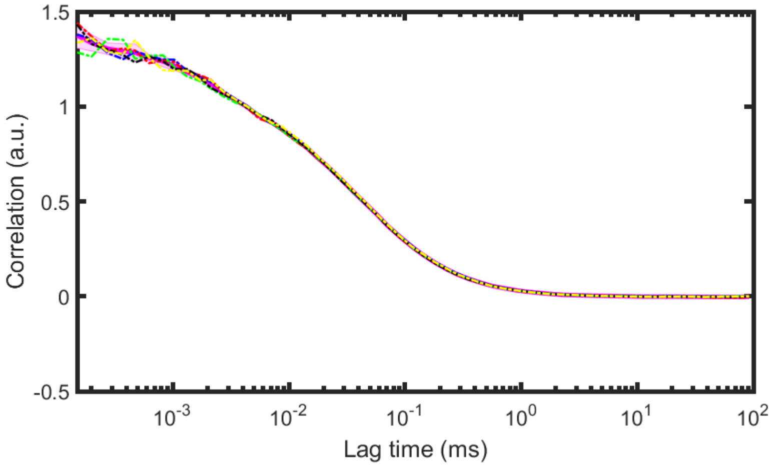

Figure S11. Fluorescence autocorrelation curves of ATTO488. FCS measurements were taken *of ATTO 488 (10 nM) diffusing in a confocal volume. The blue dotted line is repeat 1, red dotted line is repeat 2, green dotted line is repeat 3, black dotted line is repeat 4, and yellow dotted line is repeat 5. The solid pink line is the mean, with the shaded pink region showing the standard error. ATTO488, with an average diffusion time of 0.04 ms (N=5) was used to calibrate the confocal volume (0.6 fL).*

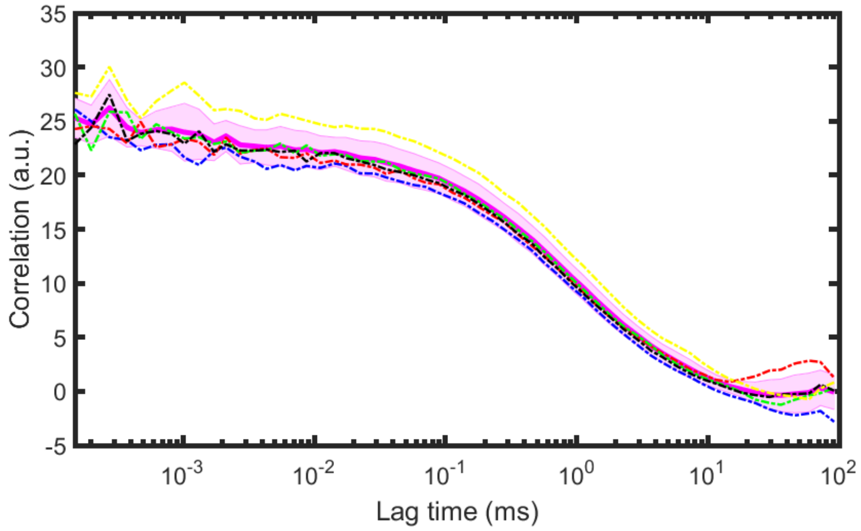

Figure S12. Fluorescence autocorrelation curves of 30 nm beads. FCS measurements were taken *of 30 nm beads diffusing in a confocal volume. The blue dotted line is repeat 1, red dotted line is repeat 2, green dotted line is repeat 3, black dotted line is repeat 4, and yellow dotted line is repeat 5. The solid pink line is the mean, with the shaded pink region showing the standard error. Beads of a known hydrodynamic size of 30 nm (quoted by the supplier) and an average diffusion time of 0.9 ms (N=3) were used to calculate the hydrodynamic size of the Qdot-Abs.*

5.1 Labelling with Qdot 525-Ab

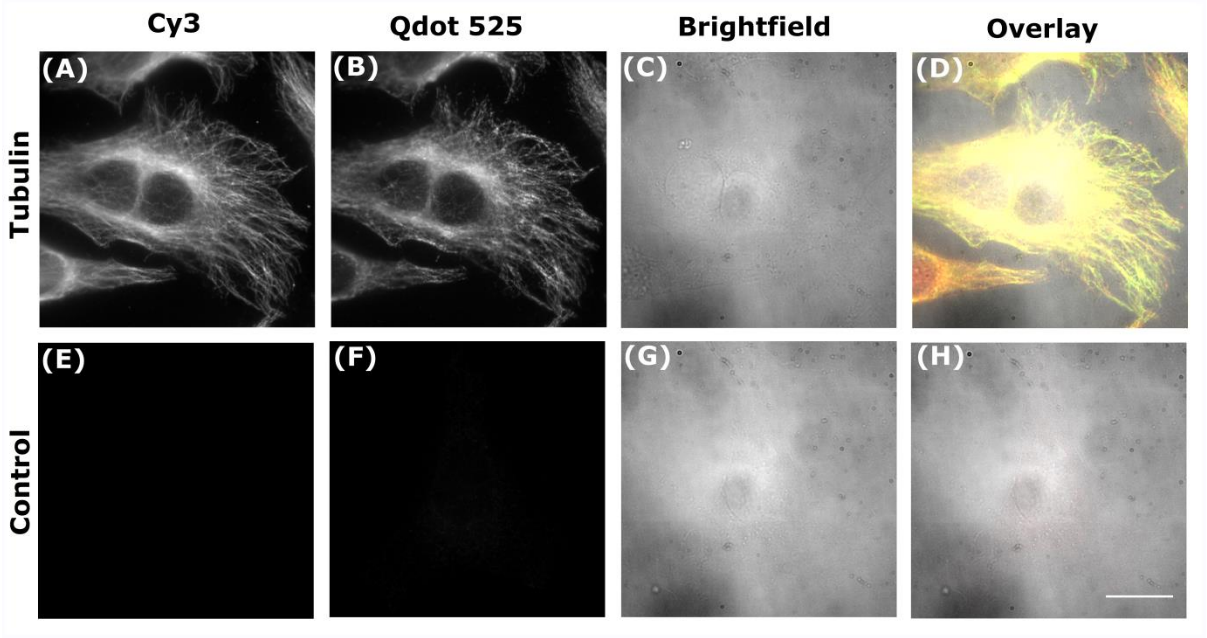

Figure S13: Labelling of tubulin with Qdot 525. Fixed HeLa cells were dual labelled with red Cy3 (A) and green Qdot 525 (B). A corresponding brightfield image of HeLa cells (C) was also taken to produce an overlaid wide-field image (D). A negative control of HeLa cells incubated simultaneously with red Cy3 (E) and green Qdot 525 (F), without the addition of a primary antibody, was done. The corresponding brightfield (G) and overlay image (H) is shown. Scale bar is 20 µm.

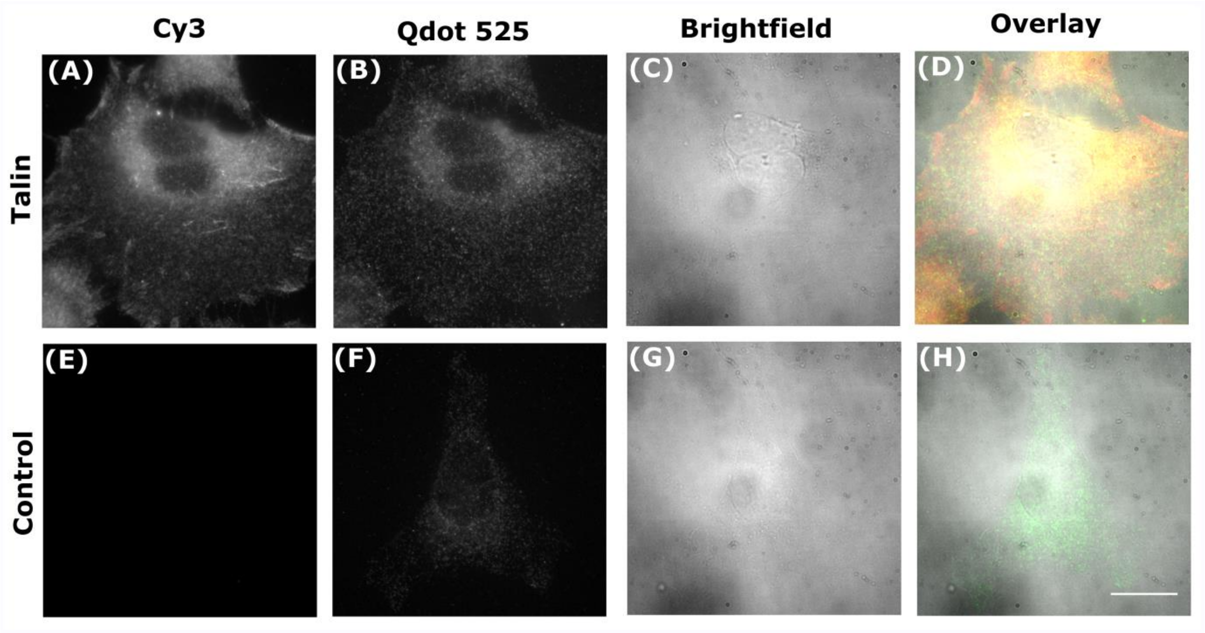

Figure S14: Labelling of talin with Qdot 525. Fixed HeLa cells were dual labelled with red Cy3-Ab (A) and green Qdot 525 (B). A corresponding brightfield image of HeLa cells (C) was also taken to produce an overlaid wide-field image (D). A negative control of HeLa cells incubated simultaneously with red Cy3 (E) and green Qdot 525 (F), without the addition of a primary antibody, was done. The corresponding brightfield (G) and overlay image (H) is shown. Scale bar is 20 µm.

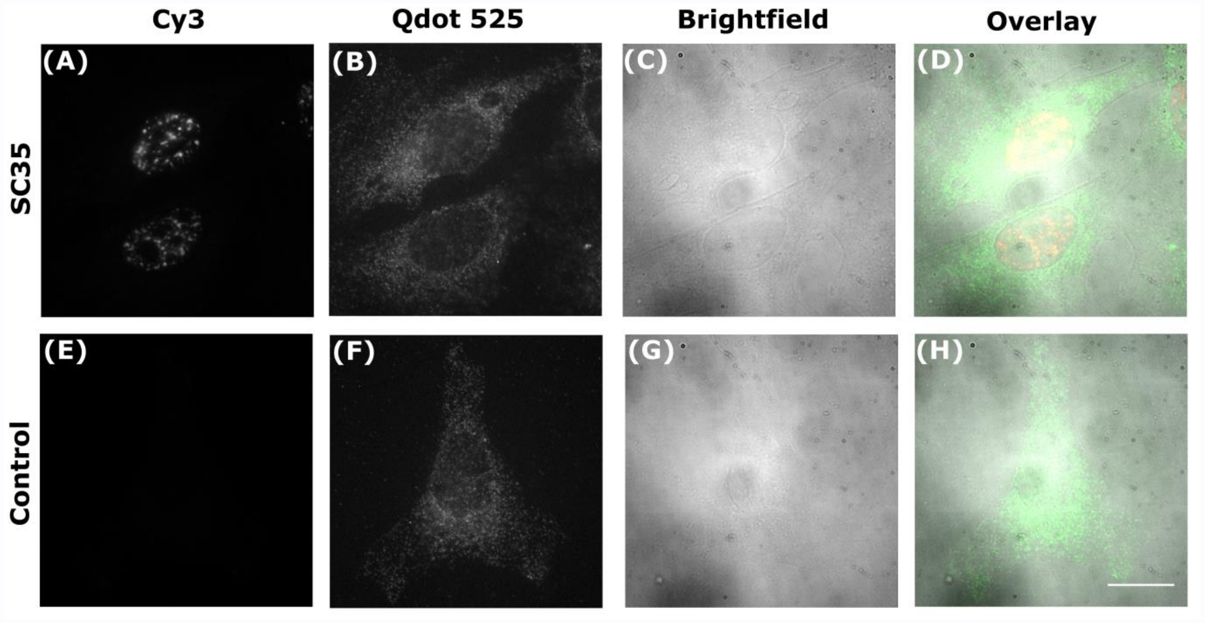

Figure S15: Labelling of SC35 with Qdot 525. Fixed HeLa cells were dual labelled with red Cy3-Ab (A) and green Qdot 525 (B). A corresponding brightfield image of HeLa cells (C) was also taken to produce an overlaid wide-field image (D). A negative control of HeLa cells incubated simultaneously with red Cy3 (E) and green Qdot 525 (F), without the addition of a primary antibody, was done. The corresponding brightfield (G) and overlay image (H) is shown. Scale bar is 20 µm.

6.1 Non-transfected controls

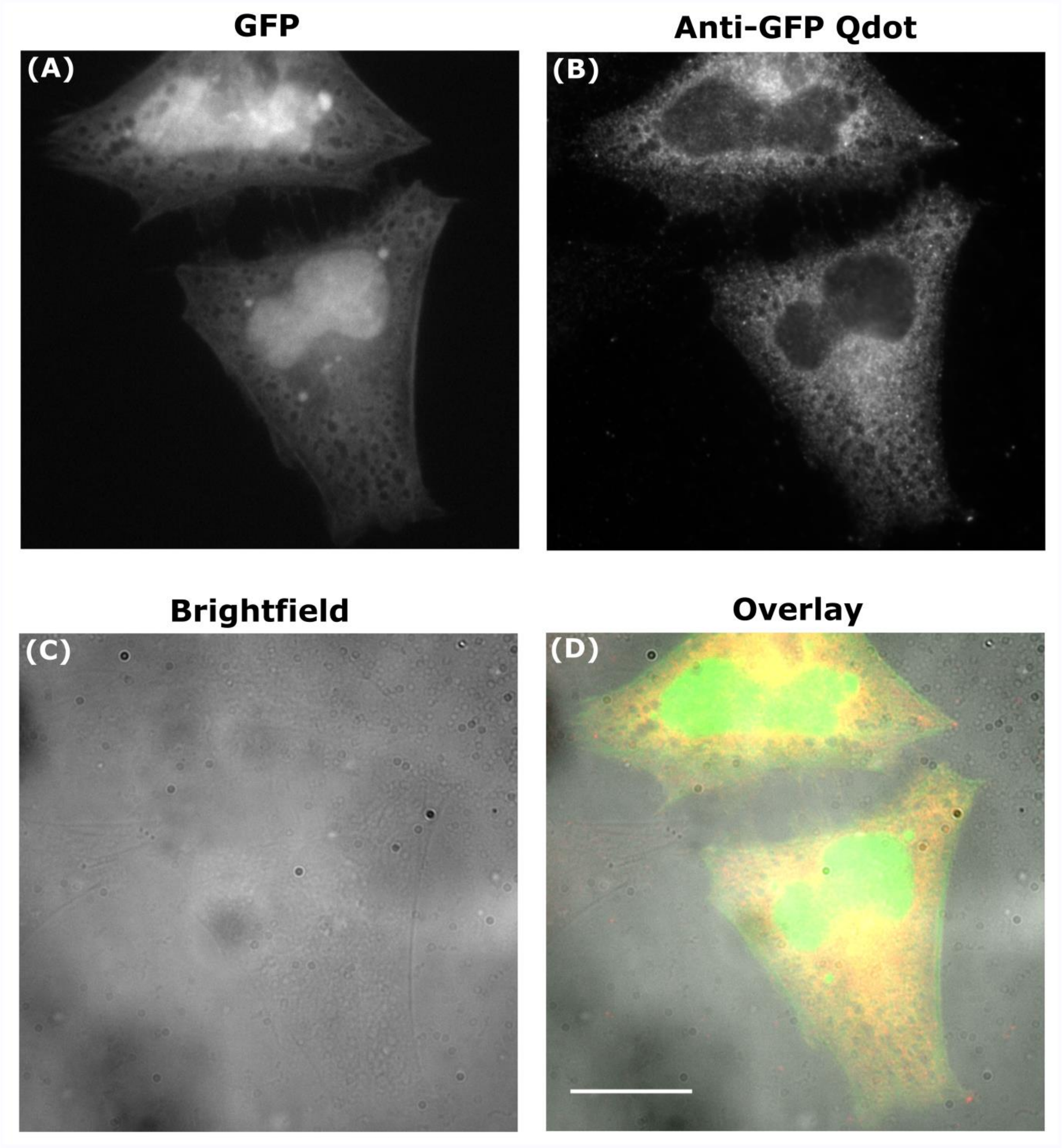

Figure S16. Qdot-Abs are unable to access the cell nucleus. Fixed HeLa cells were transfected with green unconjugated soluble GFP (A), incubated with a red anti-GFP Qdot 625 conjugate (B). A brightfield image was taken (C) and an overlay (D) to show both transfected and non-transfected cells. Scale bar is 20 µm.

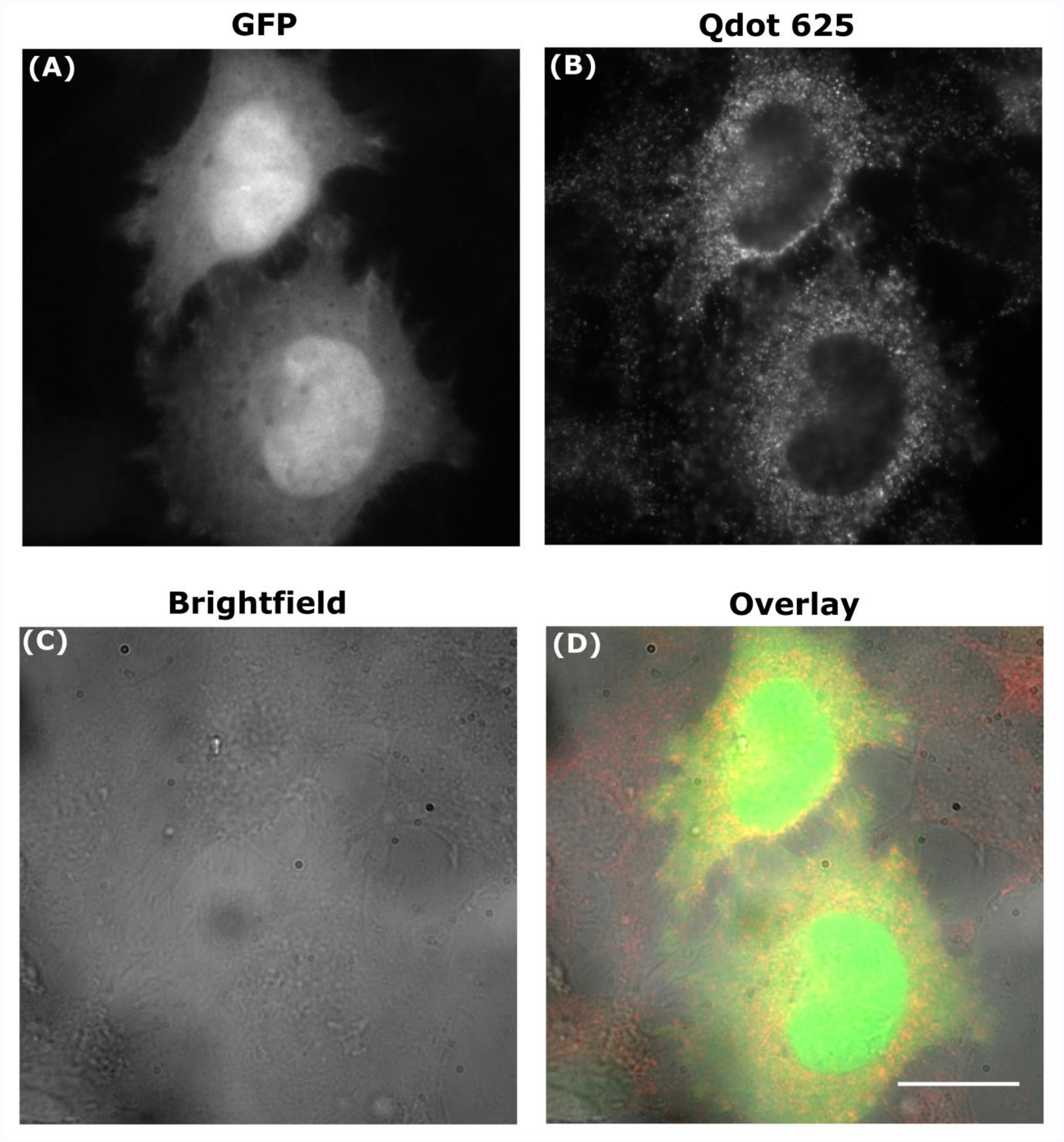

Figure S17. Qdot-Abs are unable to access the cell nucleus. Fixed HeLa cells were transfected with green unconjugated soluble GFP (A), incubated with a primary anti-GFP antibody, and red Qdot 625 conjugated secondary antibody (B). A brightfield image was taken (C) and an overlay (D) to show both transfected and non-transfected cells. Scale bar is 20 µm.

7.1 Qdot labelling summary table

Fluorescent probe	Specific binding to protein of interest		Corresponding figure(s)
Alexa Fluor 488	Fibronectin	✓	Figure 2A
	Tubulin	✓	Figure 3A and Figure S4A
	Talin	✓	Figure 4A and Figure S5A
	SC35	✓	Figure 5A and Figure S6A
Cy3	Tubulin	✓	Figure S7A
	Talin	✓	Figure S8A
	SC35	✓	Figure S9A
	Soluble GFP	✓	Figure 7E and Figure S10
Qdot 625-Ab	Soluble GFP	✓	Figure 7B and Figure S11
	Fibronectin	✓	Figure 2B
	Tubulin	✓	Figure 3B and Figure S4B
	Talin	X	Figure 4B and Figure S5B
	SC35	X	Figure 5B and Figure S6B
Qdot 525-Ab	Tubulin	✓	Figure S7B
	Talin	X	Figure S8B
	SC35	X	Figure S9B
Qdot 625-Tubulin	Tubulin	✓	Figure S3A
Qdot 625-GFP	Soluble GFP	✓	Figure 7H
	Cells expressing Tubulin-GFP	✓	Figure 3F
Qdot-Streptavidin	Tubulin	✓	Figure S3C
	EGFP-HIF2α	X	Figure 6B

Table S1. Summary of protein specific binding using different commercial antibody conjugates. Each antibody conjugate was checked as to whether it specifically bound to the protein of interest (✓) or instead non-specifically bound to cells (X). Corresponding figures where the result can be found is supplied.

References:

1. Tiwari, D,K.; Tanaka, S.; Inouye, Y.; Yoshizawa, K.; Watanabe, T,M. Sensors **2009**, 9, 9332-9354.
